# Comparison of Indocyanine Green with conventional tracers for sentinel lymph node biopsy in breast cancer: A multidisciplinary evaluation of clinical effectiveness, safety, organizational and economic impact

**DOI:** 10.1371/journal.pone.0309336

**Published:** 2024-08-29

**Authors:** Maria Pinelli, Chiara Gerardi, Emanuele Lettieri, Madalina Maioru, Laura Marone, Lorenzo Bertoldi, Giuseppe Navanteri, Maurizio Costantini, Claudio Botti, Francesca Pellini

**Affiliations:** 1 Department of Management, Economics and Industrial Engineering, Politecnico di Milano, Milan, Italy; 2 Istituto Di Ricerche Farmacologiche Mario Negri IRCCS, Milano, Italy; 3 Oncology Department Azienda Ospedaliera Universitaria Integrata (AOUI) Verona, Complex Operative Unit (UOC) Breast Surgery, Breast Unit, Verona, Italy; 4 Clinical Engineering (*IRCCS Istituto Nazionale Tumori Regina Elena—Roma*, *Italy)*, Roma, Italy; 5 Department Breast Surgery *(IRCCS Istituto Nazionale Tumori Regina Elena—Roma*, *Italy)*, Roma, Italy; Xijing Hospital, Air Force Medical University, CHINA

## Abstract

**Background:**

Breast cancer is a global health problem, and sentinel lymph node biopsy (SLNB) is the standard procedure for early-stage breast cancer. Technetium-99 (TC-99), alone or combined with blue dye (BD) are conventional tracers for SLNB, but they have safety, availability, and cost limitations. Indocyanine green (ICG) is an alternative tracer that has been gaining acceptance among healthcare professionals. This study aimed at assessing the clinical and economic value of ICG in hospital settings, using the health technology assessment (HTA) framework.

**Methods:**

We conducted a comprehensive evaluation of ICG for SLNB, based on literature sources and data collected from two Italian hospitals that switched from TC-99 to ICG. We analyzed ICG’s technical attributes through technology documentation and relevant databases. We performed a systematic literature review of 36 studies to assess the clinical effectiveness and safety of ICG. We obtained organizational insights from clinicians and the clinical engineer involved in the study. We applied Time-Driven Activity-Based Costing (TDABC) and Budget Impact Analysis (BIA) to estimate the economic impact of ICG. The ethical, legal, and social implications of ICG were considered through clinicians’ inputs and technology documentation.

**Results:**

Our results showed that ICG had equivalent or superior clinical effectiveness compared to TC-99 and BD, with minimal adverse events. ICG simplified the surgical pathways, by streamlining procedures, reducing waiting times, and increasing flexibility in scheduling surgeries. Moreover, the TDABC analysis showed significant cost reductions by avoiding the need for pre-operative lymphoscintigraphy and hospitalization, with average savings per single care pathway of around 18% for ICG compared to TC-99. Finally, ICG improved patient experience, and proved regulatory compliance.

**Conclusions:**

This study provided strong evidence for ICG’s clinical and economic value for SLNB in breast cancer. It ascertained ICG as a valuable alternative to conventional tracers, ensuring clinical effectiveness along with economic and organizational benefits.

## Introduction

Breast cancer represents one of the most diagnosed cancers worldwide [1]. Sentinel lymph node biopsy (SLNB) is currently considered the standard procedure for managing early-stage breast cancer and can help circumvent the need for a full axillary dissection [2,3].

Hence, the methods enabling precise identification of this lymph node carry significant importance in ensuring accurate oncological staging.

The current gold standard for identifying sentinel lymph nodes (SLNs) are the radioisotope Technetium-99 labeled colloids (TC-99), alone or in combination with methylene blue, which represents the previous gold standard to this aim.

However, these two techniques present disadvantages. Methylene blue a significant leakage rate, extended visualization duration, and difficulties in choosing the incision location [4,5]. TC-99, on the other hand, involves the risk of radiation exposure and the main logistical problem of requiring a Nuclear Medicine department [3].

In the last years, the use of IndoCyanine Green (ICG) spread significantly in Europe as a promising dye for different tumors, having the potential to fix logistic problems with lower costs and shorter procedures [6], and, thus, to substitute the gold standard.

Several studies in the literature have investigated the effectiveness of this dye. However, to have a comprehensive view of the impact that the introduction of this technology can have on hospitals, it is important to evaluate and highlight the different impact areas that it addresses with a multi-disciplinary perspective [7,8], providing a support to hospitals’ stakeholders in the choice of this technology.

The most renowned methodology to assess the value of novel healthcare technologies is Health Technology Assessment (HTA), a *“multidisciplinary process that uses explicit methods to determine the value of a health technology”*.

Being HTA a practice conducted at the regional/national level, in recent years Hospital-Based HTA (HBHTA) gained attention since it allows evaluations of healthcare technologies from a hospital perspective [9,10].

The aim of this study is to perform a multi-disciplinary evaluation of ICG as a substitute for TC-99, which is the gold standard for the detection of SLN, following the HTA methodology from the hospitals’ perspective.

## Materials and methods

Since there are not standardized guidelines to perform HBHTA [11], we performed this study evaluating the dimensions proposed in the EUnetHTA Core Model [12], the European framework for conducting HTA, from a hospital perspective. To perform this study, we assessed the impact of the technology for ICG with the involvement of the breast units of two renowned Italian hospitals, Azienda Ospedaliera Universitaria Integrata in Verona (hereafter Hospital 1) and Istituti Fisioterapici Ospitalieri in Rome (hereafter Hospital 2) to collect data from the field.

The methodology for each dimension under investigation is reported in this Section.

**Description and technical characteristics of technology**: to examine this domain we relied on clinical engineers and technical documents related to technology and we also consulted the ECRI database, a non-profit organization collecting information about medical devices.

**Clinical Effectiveness**: for the investigation of this domain, we performed a Systematic Literature Review coherent with Cochrane handbook for Systematic Reviews (Cochrane Handbook for Systematic Reviews of Interventions, Version 6.2, 2021 Julian Higgins^1^, James Thomas^2^ https://training.cochrane.org/handbook/current).

To do this, we drafted a research question through the development of a Patient, Intervention, Comparison, Outcomes (PICO) framework, to add robustness to our research [13].

The question was the following: *In patients ≥18 years eligible for Breast Surgical Procedure (early-stage breast cancer)*. *Does the fluorescence-guided surgery (standard*, *laparoscopic*, *robotic) for SLN mapping with indocyanine green (ICG) alone or in combination with the Blue Dye and/or TC-99m improve the efficacy*, *safety*, *timing and quality of life compared to the traditional surgery*?* *

Three databases were systematically searched up to July 19, 2023 for relevant studies. Specifically, the databases used were: MEDLINE (via PubMed), the Cochrane Central Register of Control Trials, and Embase. The search strategy launched combined text words and MeSH terms.

Several search approaches involving the utilization of English language keywords and the incorporation of Boolean logical operators were employed to formulate and oversee various combinations of search terms. These search terms encompassed terms such as “breast cancer”, “neoplasm breast”, “breast carcinoma”, “early-stage breast carcinoma”, “blue dye”, “methylene blue”, “technetium-99”, “radioactive colloid”, “indocyanine green”, “ICG”, “lymph note metastasis”. Hand searching was conducted and verification of cross-check between references between papers included.

This systematic review included randomized controlled trials (RTCs), prospective cohort studies (PCSs), retrospective cohort studies (RCSs) as primary research and meta-analysis, and systematic reviews as secondary evidence.

The studies that met one or more of the following exclusion criteria were considered not eligible: observational studies with a sample threshold <50 patients; non-human studies: studies not reporting relevant data on the outcomes of interest; studies not specifying the selection criteria or lacking the control group; studies not relevant to the technology under study or reporting other kinds of robotic technologies; studies in other languages than English; other types of publication (e.g., expert opinions, narrative reviews, editorials, and case reports).

The identification, selection, and data extraction phases were carried out independently by two couples of reviewers in a double-blind fashion between December 2020 and July 2023. Results and inconsistency were discussed among the reviewers until a consensus was reached.

Screening process, after duplicate removal, was conducted according to PRISMA statement with a first selection of title and abstracts and after full text reading and assessment. Then, we extracted data of each studies included in the final sample with a predefined spreadsheet (Excel 2007, Microsoft Corporation®).

The data collected included: 1) the study identifier (first author, year of publication); 2) the study design (type of study) 2) the population; 3) the treatment arms (intervention and control group); 4) the numerosity of the patients in each group; 5) the treatment primary and secondary outcomes.

We assessed the risk of bias with the New Ottawa scale for observational studies (https://www.ohri.ca/programs/clinical_epidemiology/oxford.asp), ROB1 for randomized clinical trials [14]. We report the assessment of the risk of bias in the Tables S5 and S6 in S1 File.

A total of 442 references were included from literature search.

After removing 73 duplicates, 366 studies had their titles and abstract evaluated. Of these, 240 were excluded after abstract review. This results in 126 articles eligible for full-text assessment, which can be found in S1 Table in S1 File. 31 articles were excluded because considered different comparator, 5 articles were excluded because of different population, 4 articles were excluded because of different aim, 3 articles were excluded because non-English, 16 because the full-text was not available, 4 articles were excluded because were abstract, 2 articles were excluded because were conference, 5 articles were excluded because were correspondence,13 because of duplicate results, 11 because data extraction was not possible. Finally, 32 records were included for the synthesis, and 36 studies extracted and analyzed. The articles included were published between 2002 and 2023. 28 were cohort studies and 8 were randomized control studies.

We included in our study three systematic reviews taken from our research that were the source of primary literature articles. In particular, from the systematic review of Mok et al. [15], one study was extracted (Rauch et al.[16]), from the systematic review of Ahmed et al.[17], two studies were extracted (Abe et al.[18] and Yamamoto et al. [19]) and from the systematic review of Thongvitokom et al. [20] four studies were extracted (Jung et al. [21], Mazouni et al. [22], Motomura et al. [23], Sugie et al. [24]).

**Fig 1:**
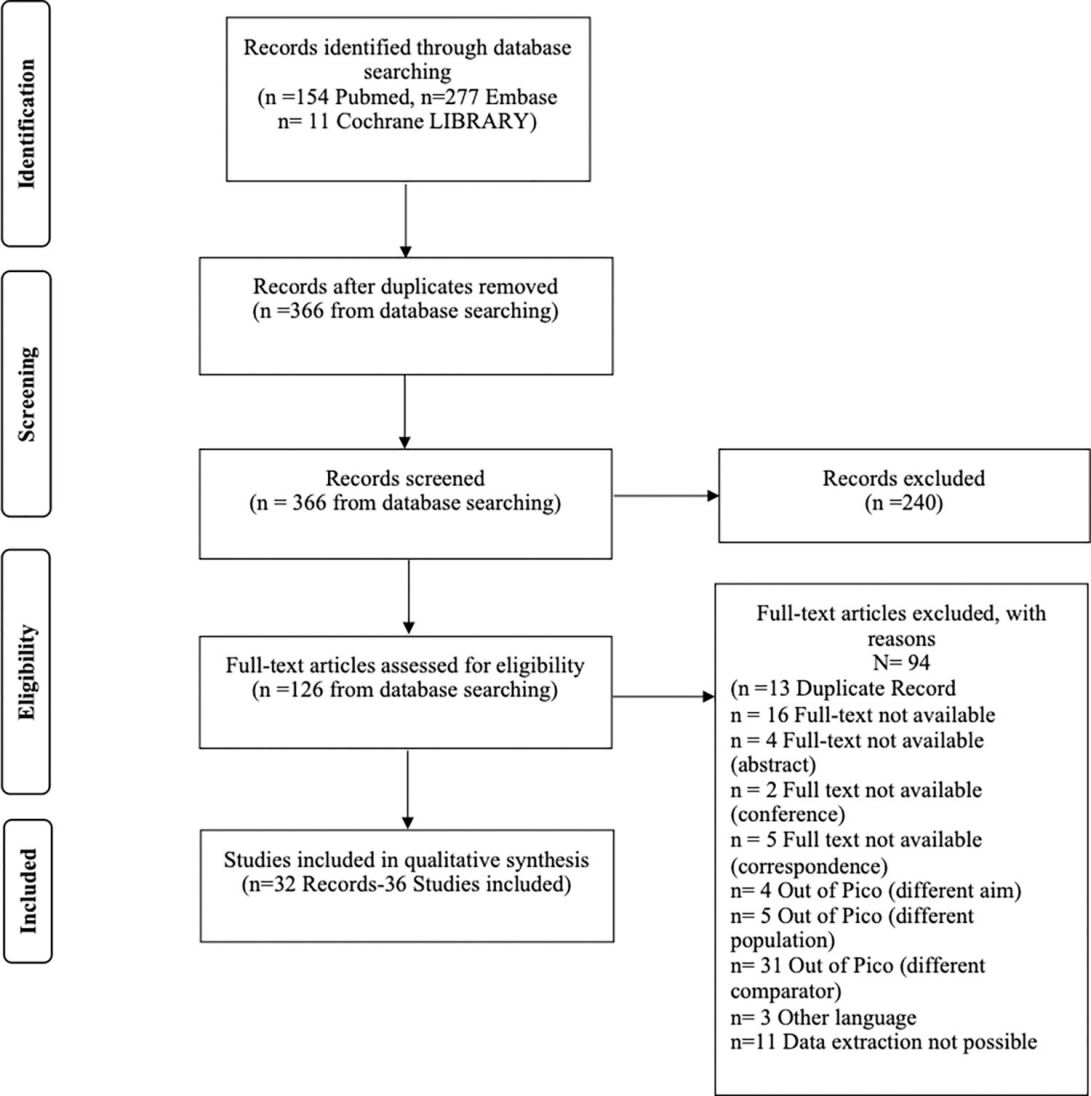
PRISMA flow diagram for search and selection of articles included in the systematic literature review.

Full process is reported with PRISMA flow-chart in Fig 1. In S1 Table, it is possible to see the PRISMA checklist.

For each article finally included, we collected information regarding: 1) the accuracy in SLN detection in terms both of *Number of patients with at least 1 SLN detected/ Total number of patients* and *Number of SLNs identified/Total number of SLNs;* 2) Other outcomes and adverse events. The detailed information regarding the characteristics of the studies included and the information collected can be found in the S2-S4 Tables in S1 File. All the data were retrieved from the selected articles.

The results of the Systematic Literature Review were validated by the clinicians involved in this study.

### Safety

This dimension was examined both through the results of the Systematic Literature Review concerning Adverse events and the investigation of the ECRI database and the MAUDE database, a repository encompassing medical device reports submitted to the FDA or voluntarily submitted by healthcare providers or patients.

### Organizational aspects

This domain has been informed by the involvement of the healthcare professionals participating to the study, who employ the technology daily. To evaluate the organizational impact, we mapped the surgical pathway of the patients object of the study and accounted for the changes between the use of ICG and other tracers like TC-99.

### Costs and economic evaluation

The economic and financial impact of the technology was assessed referring to two models of inspiration, used by Italian hospitals: Time-Driven Activity-Based Costing [25], which calculates the total cost of patient care based on the care pathway phases and the personnel involved in them, and Budget Impact Analysis [26], that determines the total economic impact taking the perspective of the hospital.

The analysis to determine the financial impact was composed by:

- Valorization of the care pathway,
- Valorization of the injection costs,
- Budget impact analysis.

The assessment was carried out starting from virtual data retrieved from the literature. Subsequently, precise and retrospective data was recovered in the two hospitals involved.

### Ethical, Legal, Social aspects (ELSA)

The ethical and social aspects were investigated through literature and opinions of the clinicians involved in the study. Concerning the legal aspects and regulatory status, both the clinicians and the documents related to the technology were consulted.

## Results

### Description and technical characteristics of technology

ICG, a tricarbocyanine and water-soluble dye originally developed by Kodak Research Laboratories for near-infrared (NIR) photography in 1955. In 1956 it received the FDA approval for diagnostic use in determining cardiocirculatory and hepatic functions, and since then it started to be used for biomedical applications. In the 1970s was used in ophthalmic angiography to demonstrate the potential of ICG to provide visual guidance in anatomical structures during surgical procedures, building a foundation for image-guided surgery [27]. From the 1980s the use of ICG in medicine has become established as standard. Recently, ICG-enhanced fluorescence was introduced in laparoscopic surgery to improve the view and guiding surgery by providing detailed anatomical information.

ICG can be injected into the human bloodstream with practically no adverse effects and it becomes fluorescent once excited with a specific light wavelength in the near infra-red (NIR) spectrum. The fluorescence can be detected using specific scopes and cameras and then transmitted to a standard monitor allowing identification of anatomical structures where the dye is present (i.e., biliary ducts, vessels, lymph nodes, etc.).

For this study, ICG is considered for the tracing of SLN in the SLNB in breast surgery procedures thanks to its broad applicability, cost-effectiveness and the possibility to be traced in real-time in high resolution. In the procedure of SLNB, it is injected into the area near the tumor and after a few minutes, the images can be captured. ICG intraoperative detection is adopted both for laparoscopic and open surgery and ECRI database reported a very high standard of performance in all settings.

A video camera and a light source (either led or halogen) emitting light with a wavelength of 760nm are sufficient to view the dye-enhanced duct or tissue. The subcutaneous lymphatic vessels become visible by fluorescence within a few minutes until the dye reaches the SLN, which in turn becomes fluorescent and can be removed. ICG is quickly eliminated from the liver in about 3 to 4 minutes.

Healthcare technology becomes pertinent when it can be compared to an existing procedure for addressing a significant healthcare problem. In this case, technical characteristics of the two main ICG comparators were examined.

TC-99 is broadly used in medical diagnostic procedures, and, as it was anticipated, it is the gold standard for the identification of SLNs. TC-99 is used extensively in nuclear medicine examinations because it is readily available and is produced using a technetium generator. TC-99 acts as a radioactive tracer and can be detected in the body by medical equipment (gamma camera).

For the localization of the SLN with the radioactive tracer TC-99, a lymphoscintigraphy examination is required. This examination is carried out in the nuclear medicine department, and after the injection of the TC-99 tracer, it is possible to visualize the accumulation of the tracer in the patient’s body using a gamma camera. The machine (gamma camera) works by capturing gamma photons from the tracer injected into the patient’s body. The device is equipped with crystals that generate visible light photons when they are hit by gamma photons. With the use of photomultipliers, the light impulses recorded in the gamma chamber are transformed into electrical impulses and reconstructed in the form of analogue images showing the distribution of TC-99 within the patient’s body in the region of the SLN. The second part of the examination is carried out with a device that uses a collimated probe to capture and count the gamma photons emitted by the patient more accurately. The information obtained from this examination supports the surgeon in identifying, removing, and analyzing the lymph node during surgery. At this stage, the surgeon uses the same device with the collimated probe, which is used in the second part of the lymphoscintigraphy examination.

Moreover, it is possible to detect the TC-99 intraoperatively using a beta-gamma detector. This device is made up of a collimated probe usually wirelessly connected to a unit. The concentration of radioactive tracer is usually returned to the surgeon both via graphical information (displayed on the device unit) and via audio, i.e a beeping sound that increases in magnitude and frequency together with the TC-99 concentration in the area of detection. Patients are usually injected with the TC-99 solution the day before surgery to provide displacement in the body and absorption of the carrying molecule by the affected organ, thus releasing the radioactivity detected by the probe.

TC-99 can be used alone or in combination with Methylene blue/Blue Dye (BD), a dye blue in colour that alone presents low effectiveness in identifying the SLNs. As ICG, it is injected at the edge of a tumor and travels through the lymph fluid to lymph nodes near the tumor. The surgeon identifies lymph nodes and lymphatic vessels with white light. Once identified, the lymph nodes that are stained with the BD are removed and are analyzed looking for cancer cells under a microscope.

### Clinical effectiveness

Effectiveness, in terms of *accuracy of sentinel lymph nodes detection rate in SLN biopsy* was investigated through the Systematic Literature Review, with the aim of understanding the differences between ICG, TC-99 and BD.

Seven studies [2,6,16,28,31], compared ICG with TC99 reporting the Sentinel nodes detection rate measured as Number of SLNs identified/Total number of SLNs. Overall, the ICG technique resulted to be as effective as TC-99. Ballardini et al. [28] in their prospective study found that the ICG method detected 99.6% (245/246) of all SLNs, while the TC-99 method detected 93.9% (231/246) of all SLNs. These results were confirmed by Samorani et al. [29] that in a prospective validation trial of 301 patients reported a sentinel node detection rate of 99% (583/589) with the ICG technique and of 77.7% (458/589) using the TC-99 method. Overall, ICG identified a higher median number of SLNs per patient than did TC-99 (1.94 SLNs vs. 1.62 SLNs). Rauch et al. [16] in their prospective study compared all the three detection methods (ICG vs TC-99 vs BD). The study showed how out of 249 SLNS detected, the majority 94% (233/249) was with ICG, 92% (230/249) with TC-99 and only 71% (176/249) with BD, concluding that ICG detected a higher rate of sentinel nodes in patients with breast cancer. The method proved to be low cost and easily handled in the clinical setting, and merits consideration as an alternative in centres without nuclear medicine facilities. Another study that confirmed ICG to have the potential to replace TC-99 as a tracer for SLNB (with the advantage of lower costs and avoidance of radioactivity) was the study by Papathemelis et al. [30] The study reported a sentinel node detection rate of 97.7% (215/220, 95% CI: 95.8–99.7%) with ICG and 78.2% (172/220, 95% CI: 72.7–83.6%) with TC-99. Five nodes (2.3%) were not visible by ICG but were detected through gamma probing of the axilla and 48 nodes (21.8%) were identified with ICG fluorescence only, showing no signal in gamma probing. In all 99 patients included in the analysis, at least one SLN was detected by TC-99 and/or ICG, meaning that SLN detection rate by dual mapping was 100%. Bargon et al. [6] in their prospective study reported that with ICG were revealed 115/125 SLNs (92.0%; 95% CI = 85.8%–96.1%), while with TC-99 were detected 107/125 SLNs (85.6%; 95% CI = 78.2–91.2). Moreover, a further analysis showed that the combination of ICG-fluorescence and 99mTc-uptake was observed in 101 sentinel lymph nodes (SLNs), representing 80.8% of the sample (95% CI = 72.8%–87.3%). In six SLNs (4.8%; 95% CI = 1.8%–10.2%), only 99mTc-uptake was detected, while 14 SLNs (11.2%; 95% CI = 6.3%–18.1%) exhibited only ICG-fluorescence. Four SLNs (3.2%; 95% CI = 0.9%–8.0%) did not contain any of the tracers. This study demonstrated that the utilization of ICG-fluorescence was as effective as 99mTc-nanocolloid. Additionally, the authors notified that performing SLNB using ICG-fluorescence was at least five times more cost-effective compared to 99mTc-nanocolloid. There are two primary reasons for this cost difference. Firstly, ICG is an affordable pharmaceutical agent. Secondly, the fluorescent camera employed for the procedure can serve other purposes, making the initial investment in equipment economically viable for hospitals.

In the study by Staubach et al. [31], among 314 SLNs that were found in 161 patients, a total of 254 SLNs (80.8%, 95% CI 71.8–89%) were identified by TC-99, compared to a total of 297 SLNs (94.6%, 95% CI 83.8–100%) identified by ICG. Finally, also the study by Dumitru et al. [2] confirmed the high sensitivity of fluorescence, which exhibits comparable performance parameters to the established gold standard. Indeed, among 154 SLNs identified, 113/154 (73.4%) were identified by TC-99 and 151/154 (98.1%) by ICG.

Other three studies [22,24], compared ICG with TC-99 reporting the detection rate measured as the number of patients with at least 1 SLN detected/ Total number of patients. Sugie et al. [24], in their prospective study, reported an overall detection rate of 97.2% (798/821 patients, p = 0.88) for ICG and 97.0% (796/821 patients) for TC-99 with a 95% CI 95.8–98.2, suggesting the ICG methods as a safe alternative to TC-99. Also the studies by Staubach et al. [31] and Dumitru et al. [2] compared these two tracers on the basis of this measure. Both the studies showed similar results: in the first one, the detection rate for TC-99 was 96.2% (155/158 patients, mean = 1.63 SLN per patient) and for ICG 94.3% (152/158 patients, mean = 1.95 SLN per patient) [31], while in the second one the detection rate for TC-99 was 97.5% (77/79 patients) while for ICG 98.7% (78/79 patients) [2].

Moreover, two studies [21,32] compared SLN detection using TC-99 alone versus TC-99 combined with ICG. The first study by Vermersch et al. [32] was a randomized controlled study of 99 patients (49 patients with TC-99, 50 patients with TC-99+ICG) that measured as a primary outcome the number of patients with fewer than two (i.e. 0 or 1) SLN detected with the ICG + TC-99 method versus the TC-99 method alone (the goal was to attain a reduction of 10% in the number of patients with fewer than two SLN identified by the use of the dual detection method to more closely approaching French recommendations to excise a mean of 2–4 SLN per patient), rather than retrieval of at least one SLN (the global definition of detection rate). It reported a detection rate of fewer than two (i.e., 0 or 1) SLN of 44.0% (22/50 patients) with the dual technique and a detection rate of 40.8% (20/49) for TC-99. This difference though, was not being statistically significant (p = 0.84). More than one SLN were detected in the remaining patients (In all patients at least one SLN was detected). The mean number of SLN identified per patient was 2.14 (SD1.23) for the dual detection group and 1.77 (SD 0.85) in the TC-99 alone group. Showing a trend toward a greater mean number of SLNB per patient in the dual detection group, although also this difference did not reach statistical significance (p = 0.09). However, the dual detection method was found to be particularly valuable when difficulties in SLN detection were encountered (As the dual detection method includes a visible component). In the second study of Jung et al. [21], The results showed that the SLN identification rate using dual method with ICG+ TC-99 was similar to that of the TC-99 only method. In addition, the identification rate for ICG alone in the dual method (DM) exceeded that for TC-99 alone, although the difference was not statistically significant (98.3% vs. 93.8%, p = 0.14).

The observational study by Somashekhar et al. [33] showed comparable performances between ICG localization and TC-99+BD, assuming that ICG can reliably replace and be employed as a sole tracer for SLNB in early breast cancer. The detection rate with ICG alone was 96% (96/100 patients), whereas, with TC-99+BD, the rate was 94% (94/100 patients).

Yuan et al. [34] in their study compared TC-99+BD to ICG+BD and demonstrated that ICG can be used as a promising alternative tracer for TC-99 in SLN mapping, and when it is combined with BD in lymphangiography, it offers comparable detection rate of 99.0% (198/200) compared to the conventional lymphatic mapping strategies of TC-99+BD with a detection rate of 99.6%, (270/271). No statistically significant difference was found in the detection rate of SLN (p = 0.79). Similar results were obtained in Agrawal et al. [35], the detection rate was 97% with ICG+BD compared with 95% in TC-99+BD. The mean number of SLNs identified were 3.17 (SD 1.84), with > 1 SLN identified in 87% of patients by TC-99+BD and 2.73 (SD 1.55) mean SLNs identified per patient with > 1 SLN identified in 79% of patients by ICG+BD. There was no statistically significant difference between the two groups for detection rate (p = 0.72). Therefore, also this study confirmed that the ICG+BD method may be a useful alternative to TC-99+BD dye for the SLNB procedure.

Suhani et al. [36] in their study reported how ICG has the potential to become the standard of care and could significantly improve sentinel lymph node programs, especially in settings where a nuclear medicine setup is not readily available. They randomized 70 patients in two groups: 35 were treated with TC-99+BD and 35 with ICG+BD. Identification rate using TC-99+BD was 91.43% (32/35 patients, p = 0.07), whereas using ICG+BD was 100% (35/35 patients, p = 0.07). In particular, in the first group the identification rate with TC-99 was 91.43% (32/35 patients, p = 0.16), and with BD 88.57% (31/35 patients, p = 0.22), while in the second group the identification rate with ICG was 100% (35/35 patients, p = 0.16) and with BD 94.28% (33/35 patients, p = 0.22). The authors also performed a cost-analysis and, contrarily to other studies, found out that the ICG+BD method is more expensive than the TC-99+BD method, but this does not comprehend the costs for setting up the nuclear medicine.

The retrospective study of Agrawal et al. [37] compared the detection rate of BD+ICG, TC-99+BD, TC-99 alone and BD alone. The highest detection rate was reported for the BD+ICG group, and it was equal to 98% (547/557 patients (p = 0.004, with a mean per patient 3.4 (SD 1.8), p<0.001). Concerning the other groups, TC99+BD had a detection rate equal to 96% (307/320 patients (p = 0.004, with a mean per patient 2.8 (SD 1.7), p<0.001); BD had a detection rate of 94% (563/598 patients (p = 0.004, with a mean per patient 2.7 (SD 1.5), p<0.001); TC-99 had a detection rate of 93.5% (43/46 patients, (p = 0.004, with a mean per patient 2.3 (SD 1.4), p<0.001).

Another study [38] compared TC-99+ICG to TC-99+BD, demonstrating the equivalence between the two dual techniques. One group of 150 patients underwent TC-99+ICG and the other TC-99+BD. In the first group, 330/351 SLNs were found to have dual tracer (94%, p = 0.156), while 9/351 (2.6%, p = 0.845) were identified by ICG and not by TC-99 and 12/351 were found by TC-99 and not by ICG (3.4%, p = 0.606). In the second group 298/315 SLNs were identified by the dual tracer (94.6%, p = 0.156), while 8/315 (2.5%, p = 0.845) were identified by BD and not by TC-99 and 9/315 were found by TC-99 and not by BD (2.9%, p = 0.606).

The study of Jung et al.[39] evaluated the detection rate of sentinel lymph node biopsy (SLNB) by a multimodal method (MMM) using a mixture of ICG, TC-99, BD compared with TC-99 alone. SLNs were detected in all patients of both groups (100% in the MMM group and 100% in the TC-99 group).

The study by Jin et al. [40] compared, in a sample of 182 patients treated with ICG, TC-99 and BD, compared the SLN detection rate between ICG, ICG+BD, ICG+TC-99, ICG+BD+TC-99 with the detection rate of BD, TC-99 and BD+TC-99. The results were the following: for ICG only the detection rate was equal to 97.8% (178/182 patients with a mean of 4.63 SLN per patient (SD ± 2.51)); for ICG+BD the detection rate was equal to 100% (182/182 patients with a mean of 4.99 SLN per patient (SD ± 2.42)); for ICG+TC-99 the detection rate was equal to 100% (182/182 patients with a mean of 4.93 per patient (SD ± 2.41)); for ICG+BD+TC-99 the detection rate was equal to 100% (182/182 patients with a mean of 5.08 per patient (SD ± 2.41)); for only BD the detection rate was equal to 89.6% (163/182 patients with a mean of 3.12 per patient (SD ± 2.48)); for TC-99 only the detection rate was equal to 94.5% (172/182 patients with a mean of 3.30 per patient (SD ± 2.10)); for BD+TC-99 the detection rate was equal to 98.9% (180/182 patients with a mean of 4.02 per patient (SD ± 2.34)). The authors also considered that patients using ICG spent less than one third of the cost of TC-99. Thus, they concluded that of ICG, with its safety profile, cost-effectiveness, and convenience, may be considered as the preferred method when radioisotope is not available or practical to use in certain settings.

The following studies [18,41–48] focused on the comparison between ICG and BD, mostly showing the superiority of the ICG technique. In particular, Guo et al. [41] in their study reported the detection rate of SLNs of 97.2% for ICG-guided SLNB, and only 81.3% for BD-guided SLNB (p < 0.05), with a mean number of SLN per patient of 3.6 SLNs in the ICG group compared to 2.1 in the BD group. Similar outcomes were found in Sugie et al. [42], reporting an SLN detection rate using the ICG fluorescence method significantly higher than that by BD method (99% vs. 78%, p < 0.001). Also, in Wang et al.[43] was reported an SLN detection rate of 100% (70/70 patients) and 93% (65/70 patients) for ICG and BD, respectively. More SLNs were detected in the ICG group (243) than in the BD group (169). The mean number of SLNs detected with ICG and BD was 3.5 (SD 1.73) and 2.4 (SD 1.49), respectively. In the study of Hojo et al. [45], ICG had a detection rate of 99.3% (140/141 patients) and its use identified a mean number of 3.8 SLNs per patient. In contrast, the BD method had a detection rate of 92.9% (105/113 patients), and its use identified on average 1.9 SLNs per patient. The study suggested that the larger number of SLNs identified with ICG could be due to the low molecular weight and a high degree of diffusion of ICG that allow it to spread beyond the SLN to secondary draining LN. The spread of the BD may be more limited.

Also the study by Coibion et al. [48] confirmed the non-inferiority of ICG compared with BD. BD alone revealed at least one SLN in 116 out of 119 patients, resulting in a detection rate of 97.5% (95% CI: 92.9%-99.1%). On the other hand, the use of ICG lead to the identification of SLNs in all 121 patients, yielding a detection rate of 100% (95% CI: 96.9%-100.0%); the mean number of SLNs detected with ICG and BD was 3.6 (SD ± 1.4) and 3.6 (SD ± 1.4), respectively.

Lastly, several studies [49–54] evaluated the combined method of ICG + BD compared to the BD method alone. Overall, the combined method achieved a higher detection rate compared to the BD alone method. Shen et al. [49], reported a detection rate of 93.3% [139/149 patients] for the BD alone group and a detection rate of 99.2% [371/374 patients] for the ICG+BD group. Therefore, the detection rate was significantly higher using the combined method of ICG+BD than that of the BD alone method (P < 0.001). Moreover, the average number of SLNs identified per patient was significantly higher in the combined method group than in the BD alone group (3.7 (SD 2.4) and 3.2 (SD 1.6), respectively; P = 0.004). Qin et al.[50] instead, didn’t show a statistically significant difference (P = 0.362) between the detection rate of SLN in the ICG + BD group 100% (60/60 patients) and the one in the BD group 96.7% (58/60 patients). Anyway, the combined method resulted to be superior to BD in terms of the mean number of SLNs detected per patient (3.4 SD1.4 (range, 2–8) vs 1.7 SD0.7 (range, 0–3) in ICG+BD and BD groups, respectively (P < 0.001), which lead to enhance accuracy and reduce false-negative rate in SLN. In the study of Zhang et al. [53] in the combination group (BD + ICG), SLNs were detected in 96.9% of patients (191/197 patients), and the average number of SLNs per patient was found to be 3.0 (range 1–6). On the other hand, in the BD group, the detection rate was 89.7% (196/218 patients), with a mean of SLNs per patient equal to 2.1 (range 1–4). This detection rate was significantly lower compared to the combination group (p = 0.004). In particular, in the combination group, the ICG only detection rate was 95.4% (188/197 patients), while the BD only detection rate was 89.3% (176/197 patients). Yang et al. [54] showed that the ICG+BD group had a 98.5% detection rate (134/136 patients), with a mean of 3.1 (± SD 0.9), while the BD group had a 91.5% detection rate (150/194 patients), with a mean of 2.6 (± SD 1.1). In particular, in the combination group 421 SLNs were found, of which 412 SLNs were detected by ICG, and 359 were detected by BD. The study didn’t show statistical difference in the number of SLNs detected between the combination group the ICG alone group. However, ICG had a better tracing effect than MB and, moreover, ICG combined with MB also increased the efficiency.

### Safety

Through the Systematic Literature Review it was possible to identify three main studies reporting data regarding adverse events of ICG compared to TC-99 or BD. Vermersch et al.[32], reported numerical evaluation of adverse events (in terms of pain, hematoma at the operative site, seroma and allergic reaction) observed since surgery until the post-operative visit. It showed how 4% of patients (2/50) experienced pain with the combined method (ICG+TC-99) and 4.1% of patients (2/ 49) experienced pain with the TC-99 alone method, p = 1.0. Hematoma at the operative site was observed in 10.0% of patients (5/50) for the combined method (ICG+ TC-99) and in 14.3% of patients (7/49) for TC-99, p = 0.55. Seroma was present in 22% of patients (11/50) for the combined method (ICG+TC-99) and 12.2% of patients (6/4) for TC-99, p = 0.29. Anyway, the two groups did not differ significantly regarding the incidence of adverse events. No patient experienced an allergic reaction related to ICG.

Nguyen et al. [38] reported results regarding number of patients with intraoperative anaphylaxis and number of patients with skin tattooing. No adverse reactions were denoted in the ICG+TC-99 group of patients, while in the BD-TC-99 cohort, 2.66% of patients (4/150) reported negative reactions that were associated with BD (2/150 intraoperative anaphylaxis and 2/150 patients with skin tattoing), p = 0.131.

In the study of Suhani et al. [36], one patient who received BD experienced blue-colored urine. Additionally, bluish skin pigmentation was observed in two patients who received TC-99+BD and three patients who received ICG+BD after breast conservation surgery. Fortunately, this pigmentation resolved in all patients except one in the ICG+BD, where it took two months to completely disappear.

### Organizational aspects

For the organizational and economic aspects ICG was compared only to TC-99, since it is the alternative practice for SLNs detection in the two hospitals involved in the study.

The adoption of ICG imaging into clinical practice would allow for an improvement in the organizational process and an optimization of the patients’ surgical pathway [3] by having a positive impact, especially on the pre-intervention phase compared with the conventional TC-99 technique.

In the traditional pathway with TC-99 the patient must perform the diagnostic exam of lymphoscintigraphy before the surgery and requires radioactive material injection at the Nuclear Medicine Department, and the consequential hospitalization. If the hospital in which the patient is supposed to be treated does not dispose of a Nuclear Medicine Department, the patient should move to the nearest available hospital to receive the radioactive injection at least 24 hours before the surgery, implying transportation and the associated costs. On the other hand, if the hospital has a nuclear medicine department, the examination can be executed there 24 hours before the surgery. In both cases, after the subcutaneous injection of TC-99, an observational time of 3 hours is required. Then the patient is discharged and returns to the hospital the day after for the surgery.

When employing the ICG for the SLN detection, the involvement of the nuclear medicine department is not necessary as the use of radioactive materials is avoided. The ICG technique indeed can be performed directly in the operating room immediately after the induction of general anaesthesia. To boost the absorption capacity of the dye, the addressed area is massaged for a few minutes. Then, a real-time lymphography is carried out by detecting the ICG fluorescence with a full HD laparoscopic laser-free near-infrared ICG system. This technology allows visualizing on the monitor the subcutaneous lymphatic route and the SLN. Afterwards, a longitudinal incision is performed. In both cases, extraction and histological examination of the SLN follow. As the tracer took a median of 4 minutes to migrate, the operating time of the two methods can be considered comparable. The surgery is also simplified by a real-time and precise guide provided to the surgeon which allows better image quality and vision when associated with fluorescence [55].

Thus, the use of ICG allows greater flexibility in surgical scheduling as the radiocolloid injection and the consequent hospitalization and observational time at the nuclear department are not necessary.

Moreover, this allows a certain degree of management in case of emergency or wrong scheduling.

Interestingly, related to this, Hospital 2 reported how, since its Breast Unit implies the Week Surgery admission to manage elective surgery, the use of ICG permits higher flexibility to schedule surgery during the whole week, even on Monday. This was not possible with TC-99 because the Nuclear Medicine Department is closed on Sunday.

Moreover, the lymphoscintigraphy exam requires a specialized team (nuclear medicine doctor, nurses, radiology technicians), the presence of a nuclear medicine department or, if absent, the transfer of the patient towards a hospital equipped with this unit and extra equipment for the acquisition of the scintigrams. In this way, the use of TC-99 requires additional personnel, skills, space, and tools, emphasizing, once again, the superiority of ICG.

### Costs and economic evaluation

As a starting point, to build economic analyses, we drafted the patients’ surgical care pathway (being composed of consultation, pre-hospitalization, surgery, lymphoscintigraphy in case of TC-99 and post-hospitalization) for three different procedures:

- Sentinel Lymph Node detection + Mastectomy + Reconstruction (Procedure A),
- Sentinel Lymph Node detection + Mastectomy (Procedure B),
- Sentinel Lymph Node detection + Tumorectomy (Procedure C).

The cost of the ICG pathway has been estimated by combining the human resources costs, given by the cost of the personnel employed along the pathway, which depends on the time actors are involved and their hourly cost, and the technology and equipment costs. The care pathway has been valorized starting from evaluating the timing associated with each phase calculated in minutes for both the ICG and TC-99 procedures.

The most influential phase is pre-hospitalization, which is on average more expensive for the TC-99 because it includes the hospitalization in the nuclear medicine department and the TC-99 machinery depreciation. For what concerns the surgery phase cost, instead, is results to be higher in the ICG case because it is comprehensive of the ICG machinery depreciation.

To quantify the cost of the personnel, the annual salary of each component involved in the process was taken as a reference. This information was retrieved from an extensive desk analysis.

Technology and equipment are analyzed considering, on the one side, the cost of the equipment, and, on the other, the costs for the surgical and observational ward. These result to be higher in the ICG case, and, for this reason, the surgery phase results more expensive using ICG.

In conclusion, the cost of the surgery employing ICG results to be greater. Nevertheless, the overall pathway shows economic advantages as the pre-hospitalization in the nuclear medicine department for the TC-99 injection is avoided.

An additional cost that was added to the previous ones to retrieve the overall cost per patient is the difference in the injection cost for the two technologies, as depicted in Tables 1 and 2. This results to be a bit higher for the ICG, due to higher consumable costs. Looking at the TC-99 injection cost, the transportation cost was considered null because, since the hospitals considered hold a nuclear medicine department, if they used TC-99, the patient would not have to travel from one hospital to another to undergo lymphoscintigraphy. This cost does not vary depending on the procedure. Also the waste disposal cost is null because the materials used for the injections are treated in the same way of the other generic reagents used in the hospital, that do not need an additional cost to be disposed.

**Table 1:** ICG injection cost.

ICG Injection cost (€/injection)	
SLN consumable (e.g., sensor)	85,00
Isotopes or molecule	23,33
Transportation cost	0
Waste disposal	-
Miscellaneous (e.g., training)	-
Total	**108,33**

**Table 2:** TC-99 injection cost.

TC-99 Injection cost (€/injection)	
SLN consumable (e.g., sensor)	0
Isotopes or molecule	100,00
Transportation cost	0
Waste disposal	-
Miscellaneous (e.g., training)	-
Total	**100,00**

In Hospital 1, the analysis has estimated an average saving of 18% for a single pathway compared to the conventional TC-99 treatment (2.146,62€ vs 2.457,65€ for SLN detection + Mastectomy + Reconstruction; 1.263,88€ vs 1.611,75€ for SLN detection + Mastectomy; and 1.099,74€ vs 1.532,61€ for SLN detection + Tumorectomy). In Hospital 2, similar results were obtained: an average saving of 17% was estimated for a single pathway compared to the conventional TC-99 treatment (2.329,96€ vs 2.659,67€ for SLN detection + Mastectomy + Reconstruction; 1.280,42€ vs 1.612,51€ for SLN detection + Mastectomy; and 1.064,90€ vs 1394,80€ for SLN detection + Tumorectomy).

As a final step, we wanted to evaluate the economic value of the ICG technique through a Budget Impact Analysis, considering the annual volume of patients and procedures, to understand the real impact on the hospital budget. The cost of the ICG equipment was depreciated over 8 years.

For Hospital 1, we considered the average annual number of patients undergoing SLN biopsy for breast surgery around 200, with a percentage of 43% for Procedure A, 12% for Procedure B and 45% for Procedure C.

For Hospital 2, we considered an average annual number of patients undergoing SLN detection for breast surgery around 816, with a percentage of 23% for Procedure A, 16% for Procedure B and 61% for Procedure C.

A 5-year projection of the economic impact was then conducted for both hospitals, starting from 2021, assuming an incremental rate of patients of 0,3% yearly in Italy (https://www.hsr.it/cancer-center/tumori/tumore-seno), and the impact of this on yearly costs. Results of this analysis is reported in Table 3 for Hospital 1 and in Table 4 for Hospital 2.

**Table 3:** Budget impact analysis Hospital 1.

ICG–Hospital 1—yearly costs					
	FY0	FY1	FY2	FY3	FY4
	*2021*	*2022*	*2023*	*2024*	*2025*
**Total Cost for Care Pathway**	313919,34	314861,10	315805,69	316753,10	317703,36
*Human resources*	*67037*,*14*	*67238*,*26*	*67439*,*97*	*67642*,*29*	*67845*,*22*
*Technologies and equipment*	*22750*,*13*	*22818*,*38*	*22886*,*84*	*22955*,*50*	*23024*,*37*
*Other*	*224132*,*07*	*224804*,*46*	*225478*,*88*	*226155*,*31*	*226833*,*78*
**Injection Cost**	21666,67	21731,67	21796,86	21862,25	21927,84
*SLN consumable (e*.*g*., *sensor)*	*17000*,*00*	*17051*,*00*	*17102*,*15*	*17153*,*46*	*17204*,*92*
*Isotopes or molecule*	*4666*,*67*	*4680*,*67*	*4694*,*71*	*4708*,*79*	*4722*,*92*
**Total annual cost (€)**	**335586,01**	**336592,77**	**337602,55**	**338615,36**	**339631,20**

**Table 4:** Budget impact analysis Hospital 2.

ICG—BREAST SETTING—yearly costs					
	FY0	FY1	FY2	FY3	FY4
	*2021*	*2022*	*2023*	*2024*	*2025*
**Total Cost for Care Pathway**	1162775,92	1166264,25	1169763,04	1173272,33	1176792,15
*Human resources*	*257775*,*46*	*258548*,*79*	*259324*,*43*	*260102*,*41*	*260882*,*71*
*Technologies and equipment*	*53339*,*76*	*53499*,*78*	*53660*,*28*	*53821*,*26*	*53982*,*73*
*Other*	*851660*,*70*	*854215*,*68*	*856778*,*32*	*859348*,*66*	*861926*,*71*
**Injection Cost**	90350,00	90621,05	90892,91	91165,59	91439,09
*SLN consumable (e*.*g*., *sensor)*	*70890*,*00*	*71102*,*67*	*71315*,*98*	*71529*,*93*	*71744*,*52*
*Isotopes or molecule*	*19460*,*00*	*19518*,*38*	*19576*,*94*	*19635*,*67*	*19694*,*57*
**Total annual cost (€)**	**1253125,92**	**1256885,30**	**1260655,95**	**1264437,92**	**1268231,23**

### Ethical, Legal, Social aspects (ELSA)

Concerning the ethical and social dimensions, the introduction of ICG has meaningful benefits compared to TC-99, both regarding time saving and, above all, connected to the care pathway.

ICG can be considered as an enabling technology to implement lean thinking and agile logic to reorganize the patient flow defining priorities while delivering the service with the same quality and safety standards [3]. In addition, by removing the lymphoscintigraphy phase it is possible to obtain a reduction on waiting lists for nuclear medicine.

Comparing ICG with TC-99 and BD it is possible to point out other advantages. One study [56] reported that while being effective in identifying the SLN, radio-colloids and BD present significant downsides. First of all, considering TC-99, radioactive signals are a significant obstacle, especially risky for pregnant women, and have a low spatial resolution. Moreover, the exposure to radioactive signals requires the patient to remain separated from family and relatives; in this sense, therefore, the use of ICG has positive effects on the patient’s quality of life. Considering BD, instead, it is characterized by secondary effects as allergic reactions or coloration and infection of the surgical area. For these reasons, ICG, using non-ionizing radiation and presenting no relevant side-effects, is an efficient alternative.

Considering legal aspects, ICG was developed during WW2 and received authorization by the Food & Drug Administration (FDA) in 1959. At first, it was mainly employed to diagnose liver functionality and subsequently in the ophthalmic and cardiological fields.

Considering the Italian regulatory context, ICG is a medical device with AIC (Autorizzazione all’Immissione in Commercio) code 971219314 and it belongs to the category of health products marked with the CE marking attesting to its compliance with European Directives 93/42/EEC, 90/385/EEC and 98/79/EC.

## Discussion and conclusion

The pursuit of the optimal technique for assessing SLNs in breast cancer patients is still controversial [57]. The aim of this study was to assess the impact of the ICG tracer for SLNs detection compared to the gold standard technology, TC-99, alone or in combination with BD. Regarding the gold standard technology, attempts to improve accuracy of TC-99 planar lymphoscintigraphy with concomitant SPECT/CT are reported in the literature. One meta-analysis focused on studies comparing SPECT/CT and planar lymphoscintigraphy and reported a significant increment in the detection rate of SLNs in the first case [58]. Other advantages reported in the article are a more precise anatomical information helpful for the surgeon leading to a change in surgical approaches, and a benefit for patients with high BMI. In addition, one of the assumed advantages of the method, i.e. obtaining a higher number of lymph nodes, is by no means proven to be better since the removal of one to three SLNs is considered to be sufficient in the current practice, consistently with the staging and not therapeutic purpose of this procedure. Moreover, the SPECT-TC method includes the increase in time and cost compared to ’standard’ lymposcintigraphy and a further increase in radiation dose. Even in case of other types of cancer, ICG proved its superiority also with respect to BD [59], maintaining residual utility in hospitals in which TC-99 or ICG cannot be accessed, or in the event of failure of these two methods as a last attempt. Moreover, some studies investigated the use of TC-99 combined with ICG [60,61], yielding favorable outcomes; however, the non-inferiority of ICG to 99mTC is demonstrated by this study, highlighting its potential future use as a single tracer.

Since healthcare professionals tend to be resistant to change [62], we wanted to provide reliable information on ICG value, not only from a clinical perspective, but having a multidisciplinary view to communicate it also to the other hospitals’ stakeholders involved in technological decision-making.

This was performed using the Health Technology Assessment methodology taking the perspective of the hospital. In particular, two hospitals where the ICG technique is used in their Breast Units were involved in this study and provided valuable information for performing the evaluation.

The clinical studies comprehended in the Systematic Literature Review conducted to assess the Clinical Effectiveness and the Safety of ICG allowed us to affirm its equivalent or even superior effectiveness compared to the gold standard, aligning with recent meta-analyses [63].

To assess the Organizational and Economic dimensions, we compared ICG only to TC-99, since it is the alternative for SLN detection in both the hospitals involved.

From an Organizational point of view, the primary benefit is the complete avoidance of the lymphoscintigraphy phase the day before surgery and this results in several advantages, in terms of time, cost, and experience, considering both the patient and the hospital point of view; in summary, this permits to manage of the entire process in a leaner and more standardized manner.

Also from an Economic perspective, ICG demonstrated to be an efficient alternative to TC-99. Our deep analysis resulted in savings mainly due to resources related to the pre-operative phase, as the lymphoscintigraphy, the observational time and the hospitalization in the nuclear department, that are avoided by employing ICG. Moreover, the cost of the Technetium-colloid is more expensive than the ICG molecule itself. Therefore, a moderate additional investment in fluorescence detecting equipment required for the use of ICG is well balanced considering the overall pathway.

Finally, from an ethical, social and legal perspective, this technology does not present downsides.

With this study we provided additional evidence regarding the multidisciplinary impact of the use of ICG in SLNB for breast cancer compared to the gold standard. Literature regarding the clinical effectiveness of this technology is rich, while studies assessing its organizational and economic impact are lacking, and this research provides a contribution in this sense.

Our analysis shows that ICG is a valuable alternative to TC-99 for SLNB, not only from a clinical point of view, but primarily from an organizational and financial perspective. These results can be considered highly relevant since the investigation was carried out by a multidisciplinary team merging both clinical and managerial backgrounds.

Further research could involve more hospitals in the analysis, in order to have a complete picture of the impact that this technology could have on a national or international level.

## Supporting information

S1 File Supporting tables. (DOCX)

S1 Table Prisma 2020 checklist. (DOCX)
