# Supplementary material for: Comparison of Indocyanine Green with conventional tracers for sentinel lymph node biopsy in breast cancer: A multidisciplinary evaluation of clinical effectiveness, safety, organizational and economic impact
Source: PLoS One. 2024 Aug 29;19(8):e0309336. doi: 10.1371/journal.pone.0309336 (PMC11361597; doi:10.1371/journal.pone.0309336)
Supplement: S1 File — (DOCX) [file pone.0309336.s001.docx]

| **Full-text identified in the literature** | | | |
| --- | --- | --- | --- |
| **Author, Year** | **Journal** | **Inclusion/Exclusion (YES/NO)** | **Reason for exclusion** |
| Shilad et al., 2018 | Ann Surg Oncol | NO | Data extraction not possible |
| Guo et al., 2017 | World J Surg Oncol | NO | Out of pico (different comparator) |
| Ersoy et al., 2018 | Clin Breast Cancer | NO | Data extraction not possible |
| Mok et al., 2019 | BJS Open | YES |  |
| Papathemelis et al., 2018 | Biomed Res Int | YES |  |
| Vaz et al., 2018 | Acta Med Port | YES |  |
| Valente et al., 2019 | J Am Coll Surg | NO | Out of pico (different comparator) |
| Liu et al., 2017 | J Int Med Res | YES |  |
| Shen et al., 2018 | J Surg Oncol | YES |  |
| Yuan et al., 2018 | Cancer Biol Med | YES |  |
| Takemoto et al., 2018 | BMC Womens Health | NO | Out of pico (different comparator) |
| Qin et al., 2019 | Ann Surg Treat Res | YES |  |
| Yuan et al., 2019 | World J Surg | NO | Out of pico (different comparator) |
| Vermersch et al., 2019 | Sci Rep | YES |  |
| Ji et al., 2017 | J Surg Res | NO | Out of pico (different comparator) |
| Grischke et al., 2015 | Geburtshilfe Frauenheilkd | NO | Out of pico (different comparator) |
| Ahmed et al., 2014 | Lancet Oncol | YES |  |
| Hokimoto et al., 2018 | Oncology | NO | Out of pico (different aim) |
| Agrawal et al., 2020 | JCO Glob Oncol | YES |  |
| Li et al., 2020 | Oncol Lett | NO | Data extraction not possible |
| He et al., 2016 | Transl Res | NO | Out of pico (different comparator) |
| Samorani et al., 2015 | Eur J Surg Oncol | YES |  |
| Murawa et al., 2009 | Br J Surg | NO | Out of pico (different population) |
| Stoffels et al., 2015 | JAMA Surg | NO | Out of pico (different aim) |
| Somashekhar et al., 2020 | Clin Breast Cancer | YES |  |
| Guo et al., 2014 | Tumour Biol | YES |  |
| Wang et al., 2020 | Gland Surg | YES |  |
| Jiang et al., 2020 | J Surg Oncol | NO | Full text not available |
| Ngô et al., 2020 | Breast J | NO | Out of pico (different comparator) |
| Liberale et al., 2020 | JMIR Res Protoc | NO | Out of pico (different population) |
| Guo et al., 2014 | World J Surg Oncol | NO | Out of pico (different comparator) |
| Takao et al., 2020 | Gan To Kagaku Ryoho | NO | Other language |
| Thongvitokomarn et al., 2020 | Asian Pac J Cancer Prev | YES |  |
| Tong et al., 2014 | J Breast Cancer | YES |  |
| Ballardini et al., 2013 | Eur J Surg Oncol | NO | Duplicated |
| Wishart et al., 2012 | Eur J Surg Oncol | NO | Out of pico (different comparator) |
| Hirano et al., 2012 | Ann Surg Oncol | YES |  |
| Inoue et al., 2016 | Breast Cancer | NO | Out of pico (different comparator) |
| Sugie et al., 2013 | Ann Surg Oncol | YES |  |
| Hojo et al., 2010 | Breast | YES |  |
| Pitsinis et al., 2017 | Ann Surg Oncol | NO | Full text not available (correspondence) |
| Jung et al., 2014 | Ann Surg Oncol | YES |  |
| Sugie et al., 2017 | Ann Surg Oncol | NO | Full text not available (correspondence) |
| Samorani et al., 2014 | Eur J Surg Oncol | NO | Full text not available (correspondence) |
| Ahmed et al., 2014 | Eur J Surg Oncol | NO | Full text not available (correspondence) |
| Ballardini et al., 2014 | Eur J Surg Oncol | NO | Full text not available (correspondence) |
| Jeremiasse et al., 2020 | European Journal of Surgical Oncology | NO | Data extraction not possible |
| York et al., 2020 | European Journal of Cancer | NO | Out of pico (different comparator) |
| Goonawardena et al., 2020 | Breast | NO | Full text not available (abstract) |
| Tegura et al., 2020 | Revue Medicale de Bruxelles | NO | Other language |
| Hashlamoun et al., 2020 | European Journal of Surgical Oncology | NO | Full text not available (abstract) |
| Weiss et al., 2020 | Radiology | NO | Full text not available (conference) |
| Ngô et al., 2020 | Breast Journal | NO | Duplicated |
| Kumar Agrawal et al., 2020 | JCO Global Oncology | NO | Duplicated |
| Chang et al., 2020 | Asian journal of surgery | NO | Out of pico (different comparator) |
| Dumitru et al., 2020 | Breast Cancer Research and Treatment | NO | Out of pico (different population) |
| Somashekhar et al., 2020 | Annals of Surgical Oncology | NO | Full text not available (conference) |
| Ngô et al., 2019 | International Journal of Gynecological Cancer | NO | Full text not available |
| Li et al., 2019 | Breast Journal | NO | Out of pico (different comparator) |
| Ali et al., 2019 | European Journal of Surgical Oncology | NO | Full text not available (abstract) |
| Kr et al., 2019 | Annals of Oncology | NO | Duplicated |
| Zhu et al., 2019 | Breast | NO | Out of pico (different comparator) |
| Kumar et al., 2019 | Annals of Surgical Oncology | NO | Out of pico (different comparator) |
| Qi et al., 2019 | Cancer Research | NO | Duplicated |
| Maeshima et al., 2019 | Cancer Research | NO | Full text not available |
| Somashekhar et al., 2019 | Cancer Research | NO | Full text not available |
| Yuan et al., 2018 | Cancer Biology and Medicine | NO | Duplicated |
| Yamagami et al., 2018 | Annals of Oncology | NO | Full text not available |
| Lopes et al., 2018 | Clinical Breast Cancer | NO | Out of pico (different population) |
| Gnanakumar et al., 2018 | European Journal of Surgical Oncology | NO | Out of pico (different comparator) |
| Krivorotko et al., 2018 | European Journal of Cancer | NO | Out of pico (different comparator) |
| Sorrentino et al., 2018 | Cancer Research | NO | Out of pico (different comparator) |
| Kern et al., 2018 | Cancer Research | NO | Full text not available |
| Ji et al., 2017 | Journal of Surgical Research | NO | Duplicated |
| Yamagami et al., 2017 | Journal of Clinical Oncology | NO | Full text not available |
| Ji et al., 2017 | Ultrasound in Medicine and Biology | NO | Out of pico (different comparator) |
| Abbaci et al., 2017 | Molecular Imaging and Biology | NO | Full text not available |
| Wichtowski et al., 2016 | European Journal of Surgical Oncology | NO | Out of pico (different population) |
| Jinno et al., 2016 | Breast Cancer | NO | Data extraction not possible |
| Benson et al., 2016 | Cancer Research | NO | Full text not available |
| Zhang et al., 2016 | Chinese Journal of Clinical Oncology | NO | Other language |
| Leitao et al., 2015 | Annals of Surgical Oncology | NO | Full text not available |
| Chi et al., 2015 | Molecular Imaging and Biology | NO | Full text not available |
| Chatterjee et al., 2015 | Cancer Journal (United States) | NO | Data extraction not possible |
| Karon et al., 2015 | Oncology Report | NO | Full text not available |
| Kou et al., 2015 | Medical Journal of Chinese People's Liberation Army | NO | Full text not available |
| Takemoto et al., 2014 | Annals of Oncology | NO | Out of pico (different comparator) |
| Ahmed et al., 2014 | European Journal of Surgical Oncology | NO | Duplicated |
| Samorani et al., 2014 | European Journal of Surgical Oncology | NO | Duplicated |
| Benson et al., 2013 | Breast Diseases | NO | Duplicated |
| Yasojima et al., 2013 | Cancer Research | NO | Full text not available |
| Verbeek et al., 2013 | Molecular Imaging and Biology | NO | Data extraction not possible |
| Verbeek et al., 2013 | European Journal of Cancer | NO | Duplicated |
| Ballardini et al., 2013 | European Journal of Cancer | YES |  |
| Benson et al., 2012 | Cancer Research | NO | Duplicated |
| Jung et al., 2012 | Cancer Research | NO | Duplicated |
| Benson et al., 2012 | European Journal of Surgical Oncology | NO | Out of pico (different comparator) |
| Schaafsma et al., 2011 | European Journal of Cancer | NO | Full text not available (abstract) |
| Sugie et al., 2011 | Journal of Clinical Oncology | NO | Full text not available |
| Wishart et al., 2011 | European Journal of Surgical Oncology | NO | Out of pico (different comparator) |
| Bleicher et al., 2010 | Breast Diseases | NO | Full text not available |
| Anan et al., 2006 | Surgery | YES |  |
| Kedrzycki et al., 2021 | Ann Surg Oncol | NO | Data extraction not possible |
| Bargon et al., 2022 | Ann Surg | YES |  |
| Hua et al., 2022 | World J Surg Oncol | NO | Out of pico (different comparator) |
| Chavda et al., 2022 | Breast Cancer Res Treat | NO | Out of pico (different comparator) |
| Tasdoven et al., 2022 | Medicine (Baltimore) | NO | Out of pico (different comparator) |
| Dumitru et al., 2022 | Ann Surg Oncol | YES |  |
| Asaga et al., 2021 | Int J Clin Oncol | NO | Out of pico (different aim) |
| Li et al., 2021 | Sci Rep | NO | Out of pico (different comparator) |
| Xu (a) et al., 2022 | Gland Surg | NO | Out of pico (different comparator) |
| Xu (b) et al., 2022 | Updates Surg | NO | Out of pico (different aim) |
| Agrawal et al., 2022 | Ecancermedicalscience | YES |  |
| Coibion et al., 2022 | Cancers (Basel) | YES |  |
| Jin et al., 2022 | Front Oncol | YES |  |
| Staubach et al., 2020 | J Cancer Res Clin Oncol | YES |  |
| Zhang et al., 2021 | Medicine (Baltimore) | YES |  |
| Yin et al., 2021 | Oncol Lett | NO | Data extraction not possible |
| Wang et al., 2021 | Front Oncol | NO | Out of pico (different comparator) |
| Liu et al., 2021 | Transl Cancer Res | NO | Data extraction not possible |
| Ou et al., 2023 | Gland Surg | NO | Out of pico (different comparator) |
| Nguyen et al., 2023 | Ann Surg Oncol | YES |  |
| Rocco et al., 2023 | Updates Surg | NO | Data extraction not possible |
| Sun et al., 2023 | Front Surg | NO | Out of pico (different comparator) |
| Yang et al., 2023 | BMC Surg | YES |  |
| Suhani et al., 2023 | World J Surg | YES |  |

Table S1: Studies identified in the literature with reason for exclusion

| **Characteristics of the included studies** | | | | | | | | |  |
| --- | --- | --- | --- | --- | --- | --- | --- | --- | --- |
| **Author, Year** | **Journal** | **Study design** | **Population** | **Study duration** | **Control group desciption** | **Intervention group description** | **Data extractors** | **Date of data extraction** |  |
| Papathemelis et al.,2018 | Biomed Res Int | Retrospective single-arm, single-center Study | Early stage Breast cancer | Between June 2016 and May 2017 | TC99 | ICG | Review team | March 2021 |  |
|  |  |  |  |  |  |  |  |  |  |
| Vaz et al.,2018 | Acta Med Port | Retrospective Study | Breast cancer | Between 2012 and 2016 | BD | ICG | Review team | March 2021 |  |
|  |  |  |  |  | TC99 |  |  |  |  |
| Liu et al.,2017 | J Int Med Res | Retrospective Study | Early stage Breast cancer | Between March 2012 and October 2013 | BD | ICG | Review team | March 2021 |  |
| Shen et al.,2018 | J Surg Oncol | Prospective non-randomized Study | T1-2 primary Breast cancer | Between January 2013 and December 2015 | BD | ICG+BD | Review team | March 2021 |  |
| Yuan et al.,2018 | Cancer Biol Med | Randomized controlled Study | Primary Breast cancer | Between March 2015 and May 2017 | TC99+BD | ICG+BD | Review team | March 2021 |  |
|  |  |  |  |  |  |  |  |  |  |
|  |  |  |  |  |  |  |  |  |  |
| Qin et al.,2019 | Ann Surg Treat Res | Prospective Randomized Study | Early stage Breast cancer | Between October 2016 and December 2017 | BD | ICG+BD | Review team | March 2021 |  |
|  |  |  |  |  | TC99 |  |  |  |  |
| Vermersch et al. ,2019 | Sci Rep | Randomized controlled Study | Breast cancer | Between April 2015 and May 2016. | TC99 | ICG+TC99 | Review team | March 2021 |  |
| Agrawal et al.,2020 | JCO Glob Oncol | Retrospective Study | Early stage Breast cancer (T1-3N0) | Between 2017 and 2019 | TC99 + BD | ICG + BD | Review team | March 2021 |  |
|  |  |  |  |  |  |  |  |  |  |
|  |  |  |  |  |  |  |  |  |  |
| Samorani et al.,2015 | Eur J Surg Oncol | Prospective validation trial. | Breast cancer | Between July 2012 and December 2013 | TC99 | ICG | Review team | March 2021 |  |
| Somashekhar et al.,2020 | Clin Breast Cancer | Prospective comparative Study | Early stage Breast cancer | Between June 2017 and December 2018. | TC99+BD | ICG | Review team | March 2021 |  |
|  |  |  |  |  |  |  |  |  |  |
| Guo et al.,2014 | Tumour Biol | Randomized clinical trial | Breast cancer | Between November 2010 and September 2012 | BD | ICG | Review team | March 2021 |  |
| Wang et al.,2020 | Gland Surg | Prospective Study | Breast cancer | Between May 2018 and October 2018 | BD | ICG | Review team | March 2021 |  |
|  |  |  |  |  |  |  |  |  |  |
| Tong et al.,2014 | J Breast Cancer | Prospective Study | Early stage Breast cancer | Between November 2010 and November 2013 | BD | ICG+BD | Review team | March 2021 |  |
|  |  |  |  |  |  |  |  |  |  |
| Yamamoto et al. ,2013 | Breast | Prospective cohort Study | Early stage Breast cancer | N.A | BD | ICG | Review team | March 2021 |  |
|  |  |  |  |  |  |  |  |  |  |
| Abe et al.,2011 | Surg Today | Prospective cohort Study | Early stage Breast cancer | Between May 2006 and August 2008 | BD | ICG | Review team | March 2021 |  |
|  |  |  |  |  |  |  |  |  |  |
| Jung et al.,2019 | *Ann Surg Oncol* | Randomized controlled Study | Breast cancer | N.A | TC99 | ICG | Review team | March 2021 |  |
|  |  |  |  |  |  |  |  |  |  |
| Mazouni et al.,2018 | Breast J | Prospective cohort Study | Breast cancer | N.A | TC99 | ICG | Review team | March 2021 |  |
| Sugie et al.,2016 | Ann Surg Oncol | Prospective single-arm, multicenter cohort Study | Breast cancer | Between May 2011 and April 2013 | TC99 | ICG | Review team | March 2021 |  |
|  |  |  |  |  |  |  |  |  |  |
| Motomura et al. ,2003 | J Surg Oncol | Prospective cohort Study | Breast cancer | N.A | TC99 | ICG | Review team | March 2021 |  |
| Ballardini et al. ,2013 | European Journal of Cancer | Prospective study | Early stage Breast cancer | Between June 2011 and January 2013 | TC99 | ICG | Review team | March 2021 |  |
| Hirano et al.,2012 | Ann Surg Oncol | Prospective study | Breast cancer | Between 2005 and 2010 | BD | BD-ICG | Review team | March 2021 |  |
| Sugie et al.,2013 | Ann Surg Oncol | Prospective multicenter Study | Breast cancer | Between February and October 2010 | BD | ICG | Review team | March 2021 |  |
| Hojo et al.,2010 | Breast | Prospective cohort Study | Breast cancer | Between August 2006 and December 2008 | BD | ICG | Review team | March 2021 |  |
|  |  |  |  |  | TC99 |  |  |  |  |
| Jung et al.,2014 | Ann Surg Oncol | Randomized open-label, single-center clinical trial study | Breast cancer | Between September 2011 and June 2012 | TC99 | TC99+BD+ICG | Review team | March 2021 |  |
|  |  |  |  |  |  |  |  |  |  |
|  |  |  |  |  |  |  |  |  |  |
|  |  |  |  |  |  |  |  |  |  |
| Anan et al.,2006 | Surgery | Prospective cohort Study | Early stage Breast cancer | Between April 2002 and March 2003 | BD | ICG | Review team | March 2021 |  |
| Rauch et al. ,2017 | Eur Surg | Prospective non-randomized Study | Early stage Breast cancer | Between February 2014 and April 2015 | TC99 | ICG | Review team | March 2021 |  |
|  |  |  |  |  |  |  |  |  |  |
|  |  |  |  |  | BD |  |  |  |  |
|  |  |  |  |  |  |  |  |  |  |
| Bargon et al., 2022 | Ann Surg | Prospective single-arm, single institution | Breast cancer (T1/T2) | Between August 2020 and June 2021 | TC99 | ICG | Review team | July 2023 |  |
| Agrawal et al., 2022 | Ecancermedicalscience | Retrospective Study | Early stage Breast cancer | Between 2011 to 2020 | BD | ICG + BD | Review team | July 2023 |  |
|  |  |  |  |  | TC99 |  |  |  |  |
|  |  |  |  |  | TC99 + BD |  |  |  |  |
| Coibion et al., 2022 | Cancers (Basel) | Prospective Randomized Study | Early stage Breast cancer | Between January 2019 and November 2020 | BD | ICG | Review team | July 2023 |  |
| Jin et al., 2022 | Front Oncol | Prospective study | Primary Breast cancer | Between March 2015 and November 2020 | BD | ICG | Review team | July 2023 |  |
|  |  |  |  |  | TC99 | ICG + BD |  |  |  |
|  |  |  |  |  | TC99 + BD | ICG+TC99 |  |  |  |
|  |  |  |  |  |  | TC99+BD+ICG |  |  |  |
| Zhang et al., 2021 | Medicine (Baltimore) | Retrospective Study | Early stage Breast cancer | Between January and December 2016 | BD | ICG + BD | Review team | July 2023 |  |
| Nguyen et al., 2023 | Ann Surg Oncol | Prospective single-centre study + retrospective | Early stage Breast cancer | Between April 2021 to October 2022 | TC99 + BD | ICG + TC-99 | Review team | July 2023 |  |
| Yang et al., 2023 | BMC Surg | Retrospective double-arm single-center study | Breast cancer | Between March 2016 to March 2020 | BD | ICG+BD | Review team | July 2023 |  |
| Suhani et al., 2023 | World J Surg | Randomized controlled Study | Early stage Breast cancer | Between 2019 to July 2022 | TC99 + BD | ICG + BD | Review team | July 2023 |  |
| Staubach et al., 2020 | J Cancer Res Clin Oncol | Retrospective double-arm single-center study | Early stage Breast cancer | Between June 2016 to May 2020 | TC99 | ICG | Review team | July 2023 |  |
| Dumitru et al., 2022 | Ann Surg Oncol | Prospective observational study | Breast cancer | Between May 2015 to October 2018 | TC99 | ICG | Review team | July 2023 |  |

Table S2: Characteristics of the included studies

| **Primary outcome - Accuracy in SLN detection** | | | | | | | | | | | | |  |
| --- | --- | --- | --- | --- | --- | --- | --- | --- | --- | --- | --- | --- | --- |
| **Author, year** | **Study design** | **Sample size** | **Intervention group description** | **Intervention group  size** | **Injection site** | **Dosing** | **Control group desciption** | **Control group size** | **Parameter** | **Scale unit** | **Value Intervention: Mean (SD)  p** | **Value control: Mean (SD)  p** |  |
| **Detection rate** | | | | | | | | | | | | |  |
| Papathemelis,2018 | Retrospective single-arm, single-center Study | 99 | ICG | 99 | Subcutaneously into the periareolar region at 3, 6, 9, and 12 o’clock | 0,5 ml of ICG (0.77 mM)  (In total, 3.33 mg) | TC99 | 99 | Detection rate | Number of patients with at least 1 SLN detected/ total number of patients (%) | Detection rate: 97/99 (98%) (95% CI 95.2%-100%) | Detection rate: 97/99 (98%) (95% CI 95.2%-100%) |  |
| Vaz,2018 | Retrospective Study | 232 | ICG | 232 | Peritumoral | 25 mg – 5 mg/mL | BD | 228 | Detection rate | Number of patients with at least 1 SLN detected/ total number of patients (%) | Detection rate: 212/232 (91.4%) ICG<BD: p=0.852 ICG<TC99: p<0.001  (95%CI 87.0%-94.6%) | Detection rate: 204/228 (89.5%)  (95%CI 85.5%-93.5%) |  |
|  |  |  |  |  |  |  | TC99 | 71 |  |  |  | Detection rate: 69/71 (97.2%)  (95% CI 90.2%-99.6%) |  |
| Liu,2017 | Retrospective Study | 60 | ICG | 60 | Subcutaneously and intradermally at the areola. | 1 ml of ICG (1 mg/ml) | BD | 60 | Detection rate | Number of patients with at least 1 SLN detected/ total number of patients (%)   Mean number of SLNs detected per patient (SD) | Detection rate: 60/60 (100%) p=0.006   mean 2.95 (SD 1.41) | Detection rate: 53/60 (88.3%)   mean 1.77 (SD 0.91) |  |
| Shen,2018 | Prospective non-randomized Study | 523 | ICG+BD | 374 | Subareolar region | 0.2 mL of ICG (1.0 mg/mL) | BD | 149 | Detection rate | Number of patients with at least 1 SLN detected/ total number of patients (%)   Mean number of SLNs detected per patient(SD) | Detection rate: 371/374 (99.2%) p<0.001  mean 3.7 (SD 2.4) p=0.004 | Detection rate: 139/149 (93.3%)  mean 3.2 (SD 1.6) |  |
| Yuan,2018 | Randomized controlled Study | 471 | ICG+BD | 200 | Intradermally into two to four spots at the same periareolar region | 1 mL of 1.25% ICG | TC99+BD | 271 | Detection rate | Number of patients with at least 1 SLN detected/ total number of patients (%)   Mean number of SLNs detected per patient (SD) | Detection rate: 198/200 (99.0%) p=0.790  mean 3.72 (SD 2.28) p=0.090 | Detection rate: 270/271 (99.6%)  mean 3.91 (SD 2.13) |  |
|  |  |  |  |  |  |  |  |  |  |  |  |  |  |
|  |  |  |  |  |  |  |  |  |  |  |  |  |  |
| Qin,2019 | Prospective Randomized Study | 180 | ICG+BD | 60 | Subdermally into the periareolar region, | 1 mL of ICG | BD | 60 | Detection rate | Number of patients with at least 1 SLN detected/ total number of patients (%)   Mean number of SLNs detected per patient (SD) | Detection rate: 60/60(100%) p=0.362  mean 3.4 (SD 1.4) p<0.001 | Detection rate: 58/60 (96.7%)  mean 1.7 (SD 0.7) |  |
|  |  |  |  |  |  |  | TC99 | 60 |  |  |  | Detection rate: 59/60(98.3%)  mean 2.4 (SD 0.7) |  |
| Vermersch,2019 | Randomized controlled Study | 99 | ICG+TC99 | 50 | Subareolar region | 2 mL of 0.5% ICG | TC99 | 49 | Detection rate | Number of patients with at fewer than two (i.e. 0 or 1) SLN detected/ total number of patients (%)   Mean number of SLNs detected per patient (SD) | Detection rate: 22/50 (44.0%) p=0.84  mean 2.14 (SD 1.23) p=0.09 | Detection rate: 20/49 (40.8%)  mean 1.77 (SD 0.85) |  |
| Agrawal,2020 | Retrospective Study | 207 | ICG + BD | 103 | Periareolar area | 1 mL of ICG (2.5 mg) | TC99 + BD | 104 | Detection rate | Number of patients with at least 1 SLN detected/ total number of patients (%)   Mean number of SLNs detected per patient (SD) | Detection rate: 100/103 (97%) p=0.72  mean 2.73 (1.55) | Detection rate: 99/104 (95%)  mean 3.17 (1.84) |  |
| Samorani,2015 | Prospective validation trial. | 301 | ICG | 301 | Subcutaneously above the tumour site | ICG (dose range, 0.4e1.2 ml) | TC99 | 301 | Detection rate | Number of patients with at least 1 SLN detected/ total number of patients (%)    Mean number of SLNs detected per patient (SD) | Detection rate: 297/301 (98.7%) (95% CI, 97.1%e99.5%) p<0.05  mean 1.94 | Detection rate: 287/301 (95.4%)  mean 1.62 |  |
| Somashekhar,2020 | Prospective comparative Study | 100 | ICG | 100 | Retro-areolar region | 3 ml of ICG | TC99+BD | 100 | Detection rate | Number of patients with at least 1 SLN detected/ total number of patients (%)   Mean number of SLNs detected per patient (SD) | Detection rate: 96/100 (96%)  mean 2.8 | Detection rate: 94/100 (94%)  mean 2.4 |  |
| Guo,2014 | Randomized clinical trial | 68 | ICG | 36 | Subareolar region | 1 ml of ICG (5 mg) | BD | 32 | Detection rate | Number of patients with at least 1 SLN detected/ total number of patients (%)   Mean number of SLNs detected per patient | Detection rate: 35/36 (97.2%)  p<0.05  mean 3.6 | Detection rate: 26/32 (81.3%)  mean 2.1 |  |
| Wang,2020 | Prospective Study | 70 | ICG | 70 | Upper outer part of the areolar | 1 ml of ICG | BD | 70 | Detection rate | Number of patients with at least 1 SLN detected/ total number of patients (%)    Mean number of SLNs detected per patient (SD) | Detection rate: 70/70 (100%)  mean 3.5 (SD 1.73) | Detection rate: 65/70 (93%)   mean 2.4 (SD 1.49) |  |
|  |  |  |  |  |  |  |  |  |  |  |  |  |  |
| Tong,2014 | Prospective Study | 169 | ICG+BD | 96 | Subareolar | 2.0-mL mixture containing ICG (10 mg) and PB (15 mg). | BD | 73 | Detection rate | Number of patients with at least 1 SLN detected/ total number of patients (%)   Mean number of SLNs detected per patient (SD) | Detection rate: 93/96 (96.9%)  p=0.005  mean 3.8 (SD 0.9) | Detection rate: 62/73 (84.9%)  mean 2.4 (SD 0.7) |  |
|  |  |  |  |  |  |  |  |  |  |  |  |  |  |
| Yamamoto et al., 2013 | Prospective cohort Study | 258 | ICG | 258 | Ultrasound scan-guided injection adjacent to SLNs | 25mg ICGin 5 mL indigo carmine blue | BD | 258 | Detection rate | Number of patients with at least 1 SLN detected/ total number of patients (%)   Mean number of SLNs detected per patient | Detection rate: 257/258 (99.6%)  mean 3.1 | Detection rate: 233/258 (90.3%)  mean 1 |  |
|  |  |  |  |  |  |  |  |  |  |  |  |  |  |
| Abe et al., 2011 | Prospective cohort Study | 128 | ICG | 128 | Areolar (intra- dermal) | 0.15 mL of 0.5% ICG solution | BD | 128 | Detection rate | Number of patients with at least 1 SLN detected/ total number of patients (%)   Mean number of SLNs detected per patient (range) | Detection rate: 128/128 (100%) p=0.0102  mean 3.1 (range 1-6) p<0.0001 | Detection rate: 84/128 (65.4%)  mean 1.0 (range 0-3) |  |
|  |  |  |  |  |  |  |  |  |  |  |  |  |  |
| Jung et al.,2019 | Randomized controlled Study | 122 | ICG+TC99 | 58 | N.A. | N.A. | TC99 | 64 | Detection rate | Number of patients with at least 1 SLN detected/ total number of patients (%)   Mean number of SLNs detected per patient | Detection rate: 57/58 (98.3%) p=0.14  mean 2.2(SD 1.13) | Detection rate: 60/64 (93.8%)  mean 1.9 (SD 1.33) |  |
|  |  |  |  |  |  |  |  |  |  |  |  |  |  |
| Mazouni et al.,2018 | Prospective cohort Study | 122 | ICG | 122 | N.A. | N.A. | TC99 | 122 | Detection rate | Number of patients with at least 1 SLN detected/ total number of patients (%) | Detection rate: 100/122 (81.9%) | Detection rate: 118/122 (96.7%) |  |
| Sugie et al.,2016 | Prospective single-arm, multicenter cohort Study | 821 | ICG | 821 | Subareolar | 1ml of 0.5% ICG | TC99 | 821 | Detection rate | Number of patients with at least 1 SLN detected/ total number of patients (%)   Mean number of SLNs detected per patient (median) | Detection rate: 798/821 (97.2%)  p=0.88  (95 % CI 95.8–98.2)  mean 2,3 (median 2,0)  p<0.001 | Detection rate: 796/821 (97%)  mean 1,7 (median 1,0) |  |
| Motomura et al., 2003 | Prospective cohort Study | 116 | ICG | 116 | N.A. | N.A. | TC99 | 116 | Detection rate | Number of patients with at least 1 SLN detected/ total number of patients (%) | Detection rate: 80/116 (69%) | Detection rate: 112/116 (96.6%) |  |
| Hirano et al., 2012 | Prospective study | 501 | BD-ICG | 108 | Subareolar | 5 mg | BD | 393 | Detection rate | Number of patients with at least 1 SLN detected/ total number of patients (%) | Detection rate: 108/108(100%)  p=0.0037 | Detection rate: 376/393(95.7%) |  |
| Sugie et al., 2013 | Prospective multicenter Study | 99 | ICG | 99 | Subareolar | 0.5–1 mL of 0.5% ICG | BD | 99 | Detection rate | Number of patients with at least 1 SLN detected/ total number of patients (%)  Mean number of SLNs detected per patient (median) | Detection rate: 98/99 (99%) p<0.001  mean 3.4 | Detection rate: 77/99 (78%)   mean 2.4 |  |
| Hojo et al., 2010 | Prospective cohort Study | 141 | ICG | 141 | Into the skin overlying the tumor and the sub-areolar region | 2 ml of ICG | BD | 113 | Detection rate | Number of patients with at least 1 SLN detected/ total number of patients (%) | Detection rate: 140/141 (99.3%) | Detection rate: 105/113 (92.9%) |  |
|  |  |  |  |  |  |  | TC99 | 28 |  |  |  | Detection rate: 28/28 (100%) |  |
| Jung et al., 2014 | Randomized open-label, single-center clinical trial study | 86 | TC99+BD+ICG | 43 | Subareolar | 0.6 mg of ICG | TC99 | 43 | Detection rate | Number of patients with at least 1 SLN detected/ total number of patients (%)    Mean number of SLNs detected per patient (SD) | Detection rate TC99+ICG+BD: 43/43(100%)  p=1 ( ICG= 43/43(100%) TC99= 43/43(100%) BD=39/43(90.7%) )  mean 3.4 (SD 1.37) | Detection rate: 43/43(100%)  mean 2.3 (SD1.04) p<0,001 |  |
|  |  |  |  |  |  |  |  |  |  |  |  |  |  |
|  |  |  |  |  |  |  |  |  |  |  |  |  |  |
|  |  |  |  |  |  |  |  |  |  |  |  |  |  |
| Anan et al., 2006 | Prospective cohort Study | 145 | ICG | 145 | Peritumoral | 2.5 mL of 1% ICG | BD | 145 | Detection rate | Number of patients with at least 1 SLN detected/ total number of patients (%)   Mean number of SLNs detected per patient (range) | Detection rate: 136/145 (93.8%)    mean 1.6 (range, 1-4)  p<.0.5 | Detection rate: 135/145 (93.1%)      mean 1.6 (range, 1-4) |  |
| Agrawal et al., 2022 | Retrospective Study | 1521 | BD+ICG | 557 | N.A. | 1 ml of ICG | BD | 598 | Detection rate | Number of patients with at least 1 SLN detected/ total number of patients (%)   Mean number of SLNs removed per patient (SD) | Detection rate: 547/557 (98%) p=0.004 Mean 3.4 (1.8) p<0.001 | Detection rate: 563/598 (94%) p=0.004 Mean 2.7 (1.5) p<0.001 |  |
|  |  |  |  |  |  |  | TC99 | 46 |  |  |  | Detection rate: 43/46 (93.5%) p=0.004 Mean 2.3 (1.4) p<0.001 |  |
|  |  |  |  |  |  |  | TC99 + BD | 320 |  |  |  | Detection rate: 307/320 (96%) p=0.004 Mean 2.8 (1.7) p<0.001 |  |
| Coibion et al., 2022 | Prospective Study | 240 | ICG | 121 | Peri-areolar level in front of the tumoral site | 0.2 ml of ICG (2 intradernal injections of 0.1 mL) | BD | 119 | Detection rate | Number of patients with at least 1 SLN detected/ total number of patients (%)  Mean number of SLNs removed per patient (± SD) | Detection rate: 121/121 (100%), (95% CI:96.9-100.0) Mean ﻿3.6 ± 1.4 | Detection rate: 116/119 (97.5%),  (95% CI: 92.9–99.1) |  |
| Jin et al., 2022 | Prospective Study | 182 | ICG | 182 | Subareolar area | 1 mL of 1.25% ICG | BD | 182 | Detection rate | Number of patients with at least 1 SLN detected/ total number of patients (%)   Mean number of SLNs removed per patient (mean ± SD) | Detection rate: 97.8% (178/182) Mean: 4.63 ± 2.51 | Detection rate: 89.6% (163/182)  Mean 3.12 ± 2.48 |  |
|  |  |  | ICG + BD | 182 |  |  | TC99 | 182 |  |  | Detection rate: 100% (182/182) Mean: 4.99 ± 2.42 | Detection rate: 94.5% (172/182) Mean: 3.30 ± 2.10 |  |
|  |  |  | ICG+TC99 | 182 |  |  | TC99 + BD | 182 |  |  | Detection rate: 100% (182/182) Mean: 4.93 ± 2.41 | Detection rate: 98.9% (180/182) Mean: 4.02 ± 2.34 |  |
|  |  |  | TC99+BD+ICG | 182 |  |  |  | 182 |  |  | Detection rate: 100% (182/182) Mean: 5.08 ± 2.41 |  |  |
| Zhang et al., 2021 | Retrospective Study | 415 | ICG + BD | 197 | Areola area | 1 mL of 1.25% ICG | BD | 218 | Detection rate | Number of patients with at least 1 SLN detected/ total number of patients (%)   Mean number of SLNs removed per patient (range) | Detection rate: 96.9% (191/197) Mean: 3.0 (range, 1-6) | Detection rate: 89.7% (196/218) Mean: 2.1 (1-4) |  |
| Yang et al., 2023 | BMC Surg | 300 | ICG + BD | 136 | Subcutaneously into the affected breast areola | 1 mL of 1.25% ICG | BD | 164 | Detection rate | Number of patients with at least 1 SLN detected/ total number of patients (%)  Mean number of SLNs removed per patient (mean ± SD) | Detection rate: 134/136 (98.5%) p=0.007 Mean = 3.1 ± 0.9 p<0.005 | Detection rate: 150/164 (91.5%) p=0.007 Mean = 2.6 ± 1.1 p<0.005 |  |
| Suhani et al., 2023 | World J Surg | 70 | ICG + BD | 35 | Peri-areolar region and peritumoral region | 3-5 mL of 1% ICG | TC99 + BD | 35 | Detection rate | Number of patients with at least 1 SLN detected/ total number of patients (%) | Detection rate: 35/35 (100%) ICG 35/35 (100%) BD 33/35 (94.28%) p=0.16, p= 0.22 | Detection rate: 32/35 (91.43%) p=0.07 TC-99 32/35 (91.43%), BD 31/35 (88.57%) p=0.16 |  |
| Staubach et al., 2020 | J Cancer Res Clin Oncol | 161 | ICG | 161 | Subcutaneously into the periareolar region at 3, 6, 9, and 12 o’clock | 0.5 mL of ICG (0.77 mM) | TC99 | 161 | Detection rate | Number of patients with at least 1 SLN detected/ total number of patients (%) Mean number of SLNs removed per patient (mean) | Detection rate: 152/158 (94.3%) Mean = 1.95 | Detection rate: 155/158 (96.2%) Mean = 1.63 |  |
| Dumitru et al., 2022 | Ann Surg Oncol | 79 | ICG | 79 | 1 mL intradermal and 1 mL subareolar | 2 mL of 0.5% ICG | TC99 | 79 | Detection rate | Number of patients with at least 1 SLN detected/ total number of patients (%) | Detection rate: 78/79 (98.7%) | Detection rate: 77/79 (97.5%) |  |
| **Sentinel nodes detection rate** | | | | | | | | | | | | |  |
| Papathemelis et al., 2018 | Retrospective single-arm, single-center Study | 99 | ICG | 99 | Subcutaneously into the periareolar region at 3, 6, 9, and 12 o’clock | 0,5 ml of ICG (0.77 mM)  (In total, 3.33 mg) | TC99 | 99 | Sentinel nodes detection rate | Number of SLNs identified/ total number of SLNs (%) | Sentinel nodes detection rate: 215/220 (97.7%)  (95%CI 95.8%-99.7%) | Sentinel nodes detection rate:172/220 (78.2%)  (95% CI 72.7%-83.6%) |  |
| Samorani et al., 2015 | Prospective validation trial. | 301 | ICG | 301 | Subcutaneously above the tumour site | ICG (dose range, 0.4e1.2 ml) | TC99 | 301 | Sentinel nodes detection rate | Number of SLNs identified/ total number of SLNs (%)  Median number of SLNs detected per patient (range) | Sentinel nodes detection rate: 583/589 (99%)  median 2 (0-5) | Sentinel nodes detection rate: 458/859(77.7%)  median 2 (0-5) |  |
| Ballardini et al., 2013 | Prospective study | 134 | ICG | 134 | Subdermally close to the tumor or into the subareolar region | 1 ml of 0.5% ICG | TC99 | 134 | Sentinel nodes detection rate | Number of SLNs identified/ total number of SLNs (%) | Sentinel nodes detection rate: 245/246 (99.6%) | Sentinel nodes detection rate: 231/246 (93.9%) |  |
| Rauch et al., 2017 | Prospective non-randomized Study | 98 | ICG | 98 | Intracutaneously in the periareolar tissue of the quadrant harboring the lesion | 2 ml of a 0.5% ICG (1mg) | TC99 | 98 | Sentinel nodes detection rate | Number of SLNs identified/ total number of SLNs (%) | Sentinel nodes detection rate: 233/249 (94%) | Sentinel nodes detection rate TC99: 230/249 (92%) |  |
|  |  |  |  |  |  |  | BD | 98 |  |  |  | Sentinel nodes detection rate BD: 176/249 (71%) |  |
| Bargon, 2022 | Prospective single-arm, single institution | 102 | ICG | 102 | Intradermally in 2 to 4 injection sites in the lateral areolar region | 2 ml of ICG | TC99 | 102 | Sentinel nodes detection rate | Number of SLNs identified/ total number of SLNs (%) | Sentinel nodes detection rate: 115/125 (92.0%; 95% CI=85.8%–96.1%) | Sentinel nodes detection rate: 107/125 (85.6%; 95% CI=78.2–91.2) |  |
| Nguyen et al., 2023 | Ann Surg Oncol | 300 | ICG+TC99 | 150 | Subdermally into the periareolar area | 1 ml of ICG (25mg/10mL) | TC99 + BD | 150 | Sentinel nodes detection rate | Number of SLNs identified/ total number of SLNs (%) | Sentinel nodes detection rate: 330/351 (94%) ICG yes and TC99 no 9 (2.6%) TC99 yes ICG no 12 (3.4%) | Sentinel nodes detection rate: 298/315 (94.6%) BD yes and TC99 no 8 (2.5%) TC99 yes and BD no 9 (2.9%) |  |
| Staubach et al., 2020 | J Cancer Res Clin Oncol | 161 | ICG | 161 | Subcutaneously into the periareolar region at 3, 6, 9, and 12 o’clock | 0.5 mL of ICG (0.77 mM) | TC99 | 161 | Sentinel nodes detection rate | Number of SLNs identified/ total number of SLNs (%) | Sentinel nodes detection rate: 297/314 (94.6%) | Sentinel nodes detection rate: 254/314 (80.8%) |  |
| Dumitru et al., 2022 | Ann Surg Oncol | 79 | ICG | 79 | 1 mL intradermal and 1 mL subareolar | 2 mL of 0.5% ICG | TC99 | 79 | Sentinel nodes detection rate | Number of SLNs identified/ total number of SLNs (%) | Sentinel nodes detection rate: 151/154 (98.1%) | Sentinel nodes detection rate: 113/154 (73.4%) |  |

Table S3: Primary outcomes of the review

| **Other outcomes** | | | | | | | | | | | | |  |
| --- | --- | --- | --- | --- | --- | --- | --- | --- | --- | --- | --- | --- | --- |
| **Author, year** | **Study design** | **Sample size** | **Intervention group description** | **Intervention group  size** | **Injection site** | **Dosing** | **Control group desciption** | **Control group  size** | **Parameter** | **Scale unit** | **Value Intervention: Mean (SD)  p** | **Value control: Mean (SD)  p** |  |
| **Sensitivity** | | | | | | | | | | | | |  |
| Papathemelis et al., 2018 | Retrospective single-arm, single-center Study | 99 | ICG | 99 | Subcutaneously into the periareolar region at 3, 6, 9, and 12 o’clock | 0,5 ml of ICG (0.77 mM)  (In total, 3.33 mg) | TC99 | 99 | Sensitivity | n/N(%) N = number of patients with tumor-positive SLNs | 21/21 (100%) | 20/21 (95.2%) (95% CI: 86.1–100%) |  |
|  |  |  |  |  |  |  |  |  | False negative rate | n/N(%) N = number of patients with tumor-positive SLNs | 0/21 (0%) | 1/21 (4.8%) |  |
| Vaz,2018 | Retrospective Study | 232 | ICG | 232 | Peritumoral | 25 mg – 5 mg/mL | BD | 228 | Sensitivity | n/N(%) N = number of patients with detected SLNs | 70/212 (33%) | 64/204 (32%) |  |
|  |  |  |  |  |  |  | TC99 | 71 |  |  |  | 22/69 (32%) |  |
| Shen,2018 | Prospective non-randomized Study | 523 | ICG+BD | 374 | Subareolar region | 0.2 mL of ICG (1.0 mg/mL) | BD | 149 | Sensitivity | n/N (%)  N=number of patients | 97/374 (26%) | 39/149 (26%) |  |
| Yuan et al., 2018 | Randomized controlled Study | 471 | ICG+BD | 200 | Intradermally into two to four spots at the same periareolar region | 1 mL of 1.25% ICG | TC99+BD | 271 | Sensitivity | n/N(%) N = number of patients with tumor-positive SLNs | 51/54 (94.4%) p=0.960 | 62/67 (92.5%) |  |
|  |  |  |  |  |  |  |  |  | False negative rate | n/N(%) N = number of patients with tumor-positive SLNs | 3/54(5.6%) | 5/67 (7.5%) |  |
|  |  |  |  |  |  |  |  |  |  |  |  |  |  |
| Qin et al., 2019 | Prospective Study | 180 | ICG+BD | 60 | Subdermally into the periareolar region, | 1 mL of ICG | BD | 60 | Sensitivity | n/N (%)  N=number of patients | 14/60 (23.3%) | 11/60(18.3%) p=0.788 |  |
|  |  |  |  |  |  |  | TC99 | 60 |  |  |  | 12/60(20%) |  |
| Somashekhar et al., 2020 | Prospective comparative Study | 100 | ICG | 100 | Retro-areolar region | 3 ml of ICG | TC99+BD | 100 | Sensitivity | n/N(%) N = number of patients with tumor-positive SLNs | 31/32 (96.8%) | 30/32 93.7% |  |
|  |  |  |  |  |  |  |  |  | False negative rate | n/N(%) N = number of patients with tumor-positive SLNs | 1/32 3.1% | 2/32 6.2% |  |
| Guo et al., 2014 | Randomized clinical trial | 68 | ICG | 36 | Subareolar region | 1 ml of ICG (5 mg) | BD | 32 | Sensitivity | n/N(%) N = number of patients with tumor-positive SLNs | 18/19 (94.7%) | 14/15 (93.3%) |  |
|  |  |  |  |  |  |  |  |  | False negative rate | n/N(%) N = number of patients with tumor-positive SLNs | 1/19 (5.3%) | 1/15 (6.6%) |  |
| Wang,2020 | Prospective Study | 70 | ICG | 70 | Upper outer part of the areolar | 1 ml of ICG | BD | 70 | Sensitivity | n/N(%) N = number of patients with detected SLNs | 25.7% (18/70) | 21.5% (14/65) |  |
|  |  |  |  |  |  |  |  |  |  |  |  |  |  |
| Tong et al., 2014 | Prospective Study | 169 | ICG+BD | 96 | Subareolar | 2.0-mL mixture containing ICG (10 mg) and PB (15 mg). | BD | 73 | Sensitivity | n/N(%) N = number of patients with tumor-positive SLNs | 28/29 | 16/18 |  |
|  |  |  |  |  |  |  |  |  | False negative rate | n/N(%) N = number of patients with tumor-positive SLNs | 1/29 (3.4%) | 2/18 (11.1%) |  |
| Mazouni et al.,2018 | Prospective cohort Study | 122 | ICG | 122 | N.A. | N.A. | TC99 | 122 | Sensitivity | n/N(%) N = number of patients with tumor-positive SLNs | 15/23 65.2% | 21/23 91.3% |  |
| Sugie et al.,2016 | Prospective single-arm, multicenter cohort Study | 821 | ICG | 821 | Subareolar | 1ml of 0.5% ICG | TC99 | 821 | Sensitivity | n/N(%) N = number of patients with tumor-positive SLNs | 168/180 (93.3%) | 162/180 (90 %) |  |
| Hirano et al., 2012 | Prospective study | 501 | BD-ICG | 108 | Subareolar | 5 mg | BD | 393 | Sensitivity | n/N (%)  N=number of patients | 16/108 (14.8 %) | 56/393 (14.2 %) |  |
| Jung et al., 2014 | Randomized open-label, single-center clinical trial study | 86 | TC99+BD+ICG | 43 | Subareolar | 0.6 mg of ICG | TC99 | 43 | Sensitivity | n/N (%)  N=number of patients | 9/43 20.9 % | 7/43 16.3 % |  |
| Coibion et al., 2022 | Prospective Study | 240 | ICG | 121 | Peri-areolar level in front of the tumoral site | 0.2 ml of ICG (2 intradernal injections of 0.1 mL) | BD | 119 | Sensitivity | n/N (%)  N=number of patients | 22/121 (18.2%) | 26/119 (21.8%) |  |
| Jin et al., 2022 | Prospective Study | 182 | ICG | 182 | Subareolar area | 1 mL of 1.25% ICG | BD | 182 | Sensitivity | n/N(%) N = number of patients with tumor-positive SLNs | 90.5% (38/42) | 83.3% (35/42) |  |
|  |  |  | ICG + BD | 182 |  |  | TC99 | 182 |  | n/N(%) N = number of patients with tumor-positive SLNs | 95.2% (40/42) | 90.5% (38/42) |  |
|  |  |  | ICG+TC99 | 182 |  |  | TC99 + BD | 182 |  | n/N(%) N = number of patients with tumor-positive SLNs | 100% (42/42) | 92.9% (39/42) |  |
|  |  |  | TC99+BD+ICG | 182 |  |  |  | 182 |  | n/N(%) N = number of patients with tumor-positive SLNs | 100% (42/42) |  |  |
| Zhang et al., 2021 | Retrospective Study | 415 | ICG + BD | 197 | Areola area | 1 mL of 1.25% ICG | BD | 218 | Sensitivity | n/N(%) N = number of patients with tumor-positive SLNs | 51/55 (92.7%) | 51/57 (89.5%) |  |
|  |  |  |  |  |  |  |  |  | False negative rate | n/N(%) N = number of patients with tumor-positive SLNs | 4/55 (7.3%) | 6/57 (10.5%) |  |
| Yang et al., 2023 | BMC Surg | 300 | ICG + BD | 136 | Subcutaneously into the affected breast areola | 1 mL of 1.25% ICG | BD | 164 | Sensitivity | n/N(%) N = number of patients with detected SLNs | 14.7% (20/134) | 9.1% (15/150) |  |
| Suhani et al., 2023 | World J Surg | 70 | ICG + BD | 35 | Peri-areolar region and peritumoral region | 3-5 mL of 1% ICG | TC99 + BD | 35 | Sensitivity | n/N (%)  N=number of patients | 10/35 (28%) p=0.40 | 7/35 (20%) p=0.40 |  |
| Staubach et al., 2020 | J Cancer Res Clin Oncol | 161 | ICG | 161 | Subcutaneously into the periareolar region at 3, 6, 9, and 12 o’clock | 0.5 mL of ICG (0.77 mM) | TC99 | 161 | Sensitivity | n/N(%) N = number of patients with tumor-positive SLNs | 43/44 (97.7%) | 41/44 (93.2%) |  |
|  |  |  |  |  |  |  |  |  | False negative rate | n/N(%) N = number of patients with tumor-positive SLNs | 1/44 (2.3%) | 3/44 (6.8%) |  |
| Dumitru et al., 2022 | Ann Surg Oncol | 79 | ICG | 79 | 1 mL intradermal and 1 mL subareolar | 2 mL of 0.5% ICG | TC99 | 79 | Sensitivity | n/N(%) N = number of patients with tumor-positive SLNs | 13/13 (100%) | 13/13 (100%) |  |
| **Pain** | | | | | | | | | | | | |  |
| Vermersch et al., 2019 | Randomized controlled Study | 99 | ICG+TC99 | 50 | Subareolar region | 2 mL of 0.5% ICG | TC99 | 49 | Pain | Number of patients with pain / total number of patients (%) | 2/50 (4.0%) p=1.0 | 2/49 (4.1%) |  |
|  |  |  |  |  |  |  |  |  | Hematoma at the operative site | Number of patients with Hematoma at the operative site / total number of patients (%) | 5 (10.0)  p=0.55 | 7 (14.3) |  |
|  |  |  |  |  |  |  |  |  | Seroma | Number of patients with Seroma at the operative site / total number of patients (%) | 11 (22.0) p=0.29 | 6 (12.2) |  |
| Nguyen et al., 2023 | Ann Surg Oncol | 300 | ICG+TC99 | 150 | Subdermally into the periareolar area | 1 ml of ICG (25mg/10mL) | TC99 + BD | 150 | Intraoperative anaphylaxis | Number of patients with intraoperative anaphylaxis / total number of patients (%) | 0 | 2 (1.3%) |  |
|  |  |  |  |  |  |  |  |  | Skin tattooing | Number of patients with Skin tattooing / total number of patients (%) | 0 | 2 (1.3%) |  |
| Suhani et al., 2023 | World J Surg | 70 | ICG + BD | 35 | Peri-areolar region and peritumoral region | 3-5 mL of 1% ICG | TC99 + BD | 35 | Blue colored urine | Number of patients with blue colored urine / total number of patients (%) | 1/70 (1,4%) | |  |
|  |  |  |  |  |  |  |  |  | Bluish skin pigmentation | Number of patients with bluish skin pigmentation / total number of patients (%) | 2/35 (5.7%) | 3/35 (8.6% |  |
| **Detection rate of metastatic SLN** | | | | | | | | | | | | |  |
| Vaz et al., 2018 | Retrospective Study | 232 | ICG | 232 | Peritumoral | 25 mg – 5 mg/mL | BD | 228 | Detection rate of metastatic SLN | mean (SD) | mean 0.42 (SD 0.66) 33% (95% CI: 26.7% - 39.8%)  ICG vs BD: p=0.110 ICG vs TC99: p=0.033 | mean 0.37 (SD 0.60) 31.4% (95% CI: 25.1% - 38.2%) |  |
|  |  |  |  |  |  |  | TC99 | 71 |  |  |  | mean 0.39 (SD 0.62) 31.9% (95% CI: 21.2% - 44.2%) |  |
| Yuan et al., 2018 | Randomized controlled Study | 471 | ICG+BD | 200 | Intradermally into two to four spots at the same periareolar region | 1 mL of 1.25% ICG | TC99+BD | 271 | Detection rate of metastatic SLN | n/N(%)  mean (SD) N = total number of SLNs | 51/200 (25.5%) p=0.510  mean 0.38 (SD 0.84) p=0.460 | 62/271 (22.9%)  mean 0.34 (SD 0.78) |  |
| Vermersch et al., 2019 | Randomized controlled Study | 99 | ICG+TC99 | 50 | Subareolar region | 2 mL of 0.5% ICG | TC99 | 49 | Detection rate of metastatic SLN | n/N(%) N = number of positive SLNs | 43/47 (91.5%) | 38/47 (80.9%) |  |
| Agrawal et al., 2020 | Retrospective Study | 207 | ICG + BD | 103 | Subcutaneously in the periareolar area | 1 mL of ICG (2.5 mg) | TC99 + BD | 104 | Detection rate of metastatic SLN | mean (SD) | mean 0.41 (SD 0.77) p=0.69 | mean 0.37 (SD 0.76) |  |
| Somashekhar et al., 2020 | Prospective comparative Study | 100 | ICG | 100 | Retro-areoral region | 3 ml of ICG | TC99+BD | 100 | Detection rate of metastatic SLN | n/N(%) N = number of positive SLNs | 42/43 (93.2%) | 40/43 (97.6%) |  |
| Abe et al., 2011 | Prospective cohort Study | 128 | ICG | 128 | Areolar (intra- dermal) | 0.15 mL of 0.5% ICG solution | BD | 128 | Detection rate of metastatic SLN | n/N (%) | 19/19 (100%) p=0.001 | 11/19 (58%) |  |
|  |  |  |  |  |  |  |  |  |  |  |  |  |  |
| Bargon et al., 2022 | Prospective single-arm, single institution | 102 | ICG | 102 | Intradermally in 2 to 4 injection sites in the lateral areolar region | 2 ml of ICG | TC99 | 102 | Detection rate of metastatic SLN | n/N(%) N = number of positive SLNs | 13/15 (86.7%; 95% CI = 59.5%–98.3%) | 12/15 (80%; 95% CI= 51.9%-95.7%) |  |
| Agrawal et al., 2022 | Retrospective Study | 1521 | BD+ICG | 557 | N.A. | 1 ml of ICG | BD | 598 | Detection rate of metastatic SLN | n/N(%) N = number of detected SLNs | 136/547 (25%) p=0.01 | 186/563 (33%) p=0.01 |  |
|  |  |  |  |  |  |  | TC99 | 46 |  | n/N(%) N = number of detected SLNs |  | 16/43 (37%) p=0.01 |  |
|  |  |  |  |  |  |  | TC99 + BD | 320 |  | n/N(%) N = number of detected SLNs |  | 99/307 (32%) p=0.01 |  |
| Jin et al., 2022 | Prospective Study | 182 | ICG | 182 | Subareolar area | 1 mL of 1.25% ICG | BD | 182 | Detection rate of metastatic SLN | n/N(%) N = number of positive SLNs |  |  |  |
|  |  |  | ICG + BD | 182 |  |  | TC99 | 182 |  | n/N(%) N = number of positive SLNs | 93.7% (74/79) |  |  |
|  |  |  | ICG+TC99 | 182 |  |  | TC99 + BD | 182 |  | n/N(%) N = number of positive SLNs |  | 86.1% (68/79) |  |
|  |  |  | TC99+BD+ICG | 182 |  |  |  | 182 |  | n/N(%) N = number of positive SLNs |  |  |  |
| Nguyen et al., 2023 | Ann Surg Oncol | 300 | ICG+TC99 | 150 | Subdermally into the periareolar area | 1 ml of ICG (25mg/10mL) | TC99 + BD | 150 | Detection rate of metastatic SLN | n/N(%) N = number of positive SLNs | 43/46 (93.5%) ICG yes TC99 no 1 (2.2%) TC99 yes ICG no 2 (4.3%) | 41/41 (100%) BD yes TC99 no 0 TC99 yes BD no 0 |  |
| **Timing** | | | | | | | | | | | | |  |
| Liu,2017 | Retrospective Study | 60 | ICG | 60 | Subcutaneously and intradermally at the areola. | 1 ml of ICG (1 mg/ml) | BD | 60 | Operation time from the beginning of ICG injection to SLNB completion ranged from 5 to 32min. | | | |  |
| Yuan,2018 | Randomized controlled Study | 471 | ICG+BD | 200 | Intradermally into two to four spots at the same periareolar region | 1 mL of 1.25% ICG | TC99+BD | 271 | the fluorescent signal was detectable within 2–5 min after ICG injection | | | |  |
| Vermersch,2019 | Randomized controlled Study | 99 | ICG+TC99 | 50 | Subareolar region | 2 mL of 0.5% ICG | TC99 | 49 | Overall duration of SLNB (min) | Mean (SD) | mean 97.7 (SD 39.3) |  |  |
| Samorani,2015 | Prospective validation trial. | 301 | ICG | 301 | Subcutaneously above the tumour site | ICG (dose range, 0.4e1.2 ml) | TC99 | 301 | The time from the injection of ICG and skin incision | Min(Median) | min 3.15 (median 4 ) |  |  |
|  |  |  |  |  |  |  |  |  | Time to remove the nodes | Min(Median) | min 5.30 (median, 10) |  |  |
| Somashekhar,2020 | Prospective comparative Study | 100 | ICG | 100 | Retro-areolar region | 3 ml of ICG | TC99+BD | 100 | The median transit time of ICG injection to fluorescence localization | Min(Range) | min 5 (range 2-20 ) |  |  |
| Jung ,2014 | Randomized open-label, single-center clinical trial study | 86 | TC99+BD+ICG | 43 | Subareolar | 0.6 mg of ICG | TC99 | 43 | Time to first SLN (min) | Mean (SD) | mean 6.5 (SD 5.16) | mean 8.0 (SD 4.35)  p=0,13 |  |
|  |  |  |  |  |  |  |  |  | Total time for SLNB (min) | Mean (SD) | mean 17.6 (SD 7.10) | mean 15.0 (SD 7.58)  p=0,09 |  |
|  |  |  |  |  |  |  |  |  | Average time for each SLN (min) | Mean (SD) | mean 6.0 (SD 3.61) | mean 7.3 (SD 3.95)  p=0,12 |  |
| Bargon, 2022 | Prospective single-arm, single institution | 102 | ICG | 102 | Intradermally in 2 to 4 injection sites in the lateral areolar region | 2 ml of ICG | TC | 102 | Time between injection and incision. | Median | Median of 7 minutes |  |  |
| Nguyen et al., 2023 | Ann Surg Oncol | 300 | ICG+TC99 | 150 | Subdermally into the periareolar area | 1 ml of ICG (25mg/10mL) | TC99 + BD | 150 | Operation duration | Mean (SD) minutes | 59 (21.5) p=0.192 | 54.6 (20.9)  p=0.192 |  |
|  |  |  |  |  |  |  |  |  | Length of stay | Mean (SD) days | 0.4 (1.1) p=0.171 | 0.8 (1.6)  p=0.171 |  |
| Suhani et al., 2023 | World J Surg | 70 | ICG + BD | 35 | Peri-areolar region and peritumoral region | 3-5 mL of 1% ICG | TC99 + BD | 35 | SLNB time | Median | 14 (6-35) | 12 (6-33) |  |

Table S4: Other outcomes of the review

| **Reference (authors, year)** |  | | | | | | |
| --- | --- | --- | --- | --- | --- | --- | --- |
|  | **BIAS** | | | | | |  |
|  | **Random sequence generation (selection bias)** | **Allocation concealment (selection bias)** | **Blinding of participants and personnel (performance bias)** | **Blinding of outcome assessment (detection bias)** | **Incomplete outcome data (attrition bias)** | **Selective reporting (reporting bias)** | **Other bias** |
| Coibion et al, 2022 | low risk | low risk | high risk | unclear risk | low risk | low risk | low risk |
| Suhani et al., 2023 | low risk | low risk | unclear risk | unclear risk | low risk | low risk | low risk |
| Yuan et al.,2018 | unclear risk | unclear risk | unclear risk | unclear risk | low risk | low risk | low risk |
| Qin et al., 2019 | unclear risk | unclear risk | unclear risk | unclear risk | low risk | low risk | low risk |
| Vermersch et al., 2019 | low risk | low risk | unclear risk | unclear risk | high risk | low risk | low risk |
| Guo et al.,2014 | unclear risk | unclear risk | unclear risk | unclear risk | low risk | low risk | low risk |
| Jung et al.,2019 | unclear risk | unclear risk | unclear risk | unclear risk | high risk | low risk | low risk |
| Jung et al., 2014 | unclear risk | unclear risk | unclear risk | unclear risk | low risk | low risk | low risk |

Table S5: Risk of bias Randomized Controlled Trials

| **Reference (authors, year)** | **BIAS-Cohort study** | | | | | | | |
| --- | --- | --- | --- | --- | --- | --- | --- | --- |
|  | **Selection** | | | | **Comparability** | **Outcome** | | |
|  | **1) Representativeness of the exposed cohort** a) truly representative of the average _______________ (describe) in the community * b) somewhat representative of the average ______________ in the community* c) selected group of users eg nurses, volunteers d) no description of the derivation of the cohort | **2) Selection of the non exposed cohort** a) drawn from the same community as the exposed cohort * b) drawn from a different source c) no description of the derivation of the non exposed cohort | **3) Ascertainment of exposure** a) secure record (eg surgical records) * b) structured interview* c) written self report d) no description | **4) Demonstration that outcome of interest was not present at start of study** a) yes * b) no | **1) Comparability of cohorts on the basis of the design or analysis** a) study controls for _____________ (select the most important factor) * b) study controls for any additional factor* (This criteria could be modified to indicate specific control for a second important factor.)  se non lo fanno -> not assessed | **1) Assessment of outcome** a) independent blind assessment *  b) record linkage * c) self report  d) no description | **2) Was follow-up long enough for outcomes to occur** a) yes (select an adequate follow up period for outcome of interest) * b) no | **3) Adequacy of follow up of cohorts** a) complete follow up - all subjects accounted for *  b) subjects lost to follow up unlikely to introduce bias - small number lost - > ____ % (select an adequate %) follow up, or description provided of those lost) * c) follow up rate < ____% (select an adequate %) and no description of those lost d) no statement |
| Jin et al., 2022 | a* | a* | a* | a* | a* | b* | a* | a* |
| Nguyen et al., 2023 | a* | a* | a* | a* | a* | b* | a* | a* |
| Bargon et al., 2022 | a* | a* | a* | a* | a* | b* | a* | a* |
| Agrawal et al., 2022 | a* | a* | a* | a* | not assessed | b* | a* | a* |
| Yang et al., 2023 | a* | a* | a* | a* | a* | b* | a* | a* |
| Staubach et al., 2020 | a* | a* | a* | a* | a* | b* | a* | a* |
| Dumitru et al., 2022 | a* | a* | a* | a* | a* | b* | a* | a* |
| Papathemelis et al.,2018 | a* | a* | a* | a* | not assessed | b* | a* | b* |
| Liu et al., 2017 | a* | a* | a* | a* | a* | b* | a* | a* |
| Shen et al., 2018 | a* | a* | a* | a* | a* | b* | a* | b* |
| Agrawal et al., 2020 | a* | a* | a* | a* | a* | b* | a* | a* |
| Samorani et al., 2015 | a* | a* | a* | a* | a* | b* | a* | a* |
| Somashekhar et al.,2020 | a* | a* | a* | a* | a* | b* | a* | a* |
| Wang et al., 2020 | a* | a* | a* | a* | a* | b* | a* | a* |
| Tong et al., 2020 | a* | a* | a* | a* | a* | b* | a* | a* |
| Yamamoto et al., 2013 | a* | a* | a* | a* | a* | b* | a* | a* |
| Abe et al., 2011 | a* | a* | a* | a* | not assessed | b* | a* | a* |
| Mazouni et al.,2018 | a* | a* | a* | a* | a* | b* | a* | a* |
| Sugie et al., 2013 | a* | a* | a* | a* | a* | b* | a* | a* |
| Motomura et al., 2003 | a* | a* | a* | a* | a* | b* | a* | a* |
| Ballardini et al.,2013 | a* | a* | a* | a* | a* | b* | a* | a* |
| Hirano et al., 2012 | a* | a* | a* | a* | a* | b* | a* | a* |
| Hojo et al., 2010 | a* | a* | a* | a* | a* | b* | a* | a* |
| Sugie et al., 2016 | a* | a* | a* | a* | a* | b* | a* | b* |
| Anan et al., 2006 | a* | a* | a* | a* | a* | b* | a* | a* |
| Rauch et al., 2017 | a* | a* | a* | a* | a* | b* | a* | a* |
| Vaz et al., 2018 | a* | a* | a* | a* | a* | b* | a* | a* |
| Zhang et al., 2021 | a* | a* | a* | a* | a* | b* | a* | a* |

Table S6: Risk of bias cohort studies
